# B cell–intrinsic CXCR3 drives efficient generation of ectopic pulmonary germinal center responses to influenza A virus infection

**DOI:** 10.1073/pnas.2535787123

**Published:** 2026-06-30

**Authors:** Timona S. Tyllis, Todd S. Norton, Caitlin Abbott, Dylan J. McPeake, Kevin A. Fenix, Jasmine J. Wilson, Ervin E. Kara, Kim L. Good-Jacobson, Mohammed Alsharifi, Shaun R. McColl, Iain Comerford

**Affiliations:** ^a^ Research Centre for Infectious Diseases, School of Biological Sciences, Adelaide University, Adelaide, SA 5005, Australia; ^b^ School of Medicine, College of Health, Adelaide University, Adelaide, SA 5000, Australia; ^c^ Medical Oncology and the Basil Hetzel Institute for Translational Health Research, The Queen Elizabeth Hospital, Central Adelaide Local Health Network, Woodville, SA 5011, Australia; ^d^ Immunity Program and Department of Biochemistry and Molecular Biology, Biomedicine Discovery Institute, Monash University, Clayton, VIC 3800, Australia

**Keywords:** pulmonary B cell responses, respiratory viral infection, ectopic germinal center, CXCR3

## Abstract

Chemotactic receptors involved in generation of ectopic pulmonary germinal centers (GCs) within inducible bronchus-associated lymphoid tissue (iBALT) are poorly defined. Here, using CIBER *Cxcr3*-reporter mice, we demonstrate that the prototypical type 1 inflammatory chemokine receptor CXCR3 is highly induced in influenza A virus (IAV)-reactive B cells in the mediastinal lymph node, spleen, lung, peripheral blood, and airways following intranasal infection. Notably, elevated *Cxcr3* was observed in ectopic pulmonary germinal center B (GCB) cells in iBALT relative to their contemporaneous counterparts in secondary lymphoid organs across the timecourse of the response to IAV infection. Mice with a B cell–specific deletion of *Cxcr3* displayed a 50 to 60% reduction in the frequency and number of ectopic GCB cells in the lungs at the peak of the response following IAV infection, relative to controls. Furthermore, in cotransfers, *Cxcr3*-deficient B cells were substantially outcompeted by their *Cxcr3*-sufficient counterparts for ectopic pulmonary GC participation, but were not impacted with respect to GCB cell frequencies in other compartments. Thus, the data elucidate the requirement of B cell–intrinsic CXCR3 expression for efficient generation of ectopic pulmonary GCB cell responses in iBALT following respiratory viral infection with IAV, a finding that broadens understanding of the molecular cues underpinning this key component of local protective humoral immunity to IAV.

## Significance

Germinal center B (GCB) cell responses in ectopic lymphoid tissues (ELTs) are critical for humoral immunity in nonlymphoid tissues. Coordinated expression of distinct migratory receptors underpins successful generation of GCB cell responses in secondary lymphoid tissues. However, migratory receptors required for generation of GCB cell responses in ELTs are poorly defined. Analyzing inducible bronchus-associated lymphoid tissue (iBALT), an archetypal ELT forming in the lung following influenza A virus (IAV) infection, we show B cell expression of CXCR3 is required for efficient generation of GCB cell responses in iBALT. These findings reveal a molecular requirement for ectopic GC generation in the lung and advance understanding of migratory receptor-mediated regulation of pulmonary B cell responses following respiratory viral infection.

The key chemoattractant receptors involved in B cell activation and induction of GCs in secondary lymphoid tissues (SLOs) are well characterized, with the chemokine receptors CCR7, CXCR4, and CXCR5, together with the lipid sensing G-protein-coupled receptors EBI2 and S1PR2, having important roles in guiding B cells through distinct microenvironmental transitions within SLOs during their differentiation journey (1–5). However, the migratory receptors underpinning B cell responses in ELTs remain poorly understood. In response to infection with respiratory pathogens, such as IAV, iBALT, an archetypal ELT, forms in the lung parenchyma (6–9), providing an important microenvironment that fosters proficient local humoral immunity (9–15). Notably, following IAV infection, responding B cells participate in iBALT-residing persistent GC reactions that yield hypermutated, cross-reactive, local memory B cells (MBCs) in the lung that confer protective immunity (12). However, the specific chemoattractant receptors supporting generation and maintenance of ectopic GCs in iBALT are poorly defined.

CXCR3 is well recognized for orchestrating many facets of T cell biology, coordinating migratory events involved in T cell priming, inflammatory tissue-homing, microanatomical positioning within lymphoid and peripheral tissues, and reactivation of memory (16–32). In contrast, while CXCR3 expression has been observed on B cells in several physiological and pathological settings in mice and humans (5, 33–46), functional regulation of B cell biology via CXCR3 is poorly understood. Although recent findings have shown CXCR3 has important roles in coordinating the biology of tissue-resident memory B (BRM) cells and IgA-producing antibody secreting cells (ASCs) in the lung in the context of IAV infection (34, 47, 48), a detailed analysis of the expression and function of CXCR3 in B cells following primary IAV infection is lacking.

Here, using “CXCR3 internal ribosome entry site bicistronic enhanced GFP reporter” (CIBER) *Cxcr3*-reporter mice (49), we provide a detailed spatiotemporal analysis of CXCR3 expression in the B cell compartment following primary respiratory viral infection, demonstrating that CXCR3 is dynamically expressed by responding B cells in multiple anatomical compartments as the response to IAV progresses in vivo. CXCR3 is most prominently expressed on ectopic GCB cells and class-switched memory B (swB_mem_) cells at the site of infection, in the lung iBALT, where it is substantially elevated compared to their respective counterparts in SLOs. Assessment of mice with B cell–specific deletion of CXCR3 revealed that CXCR3 was required by B cells for efficient generation of pulmonary GCB cell responses following IAV infection in both isolated and competitive settings. Thus, the findings position CXCR3 as an important B cell–intrinsic driver of ectopic pulmonary GCB cell responses in iBALT following IAV infection.

## Results

### Ectopic Pulmonary GCB Cells Are Enriched for CXCR3 Expression Compared to Their Counterparts in SLOs during Respiratory IAV Infection.

**Fig. 1.**
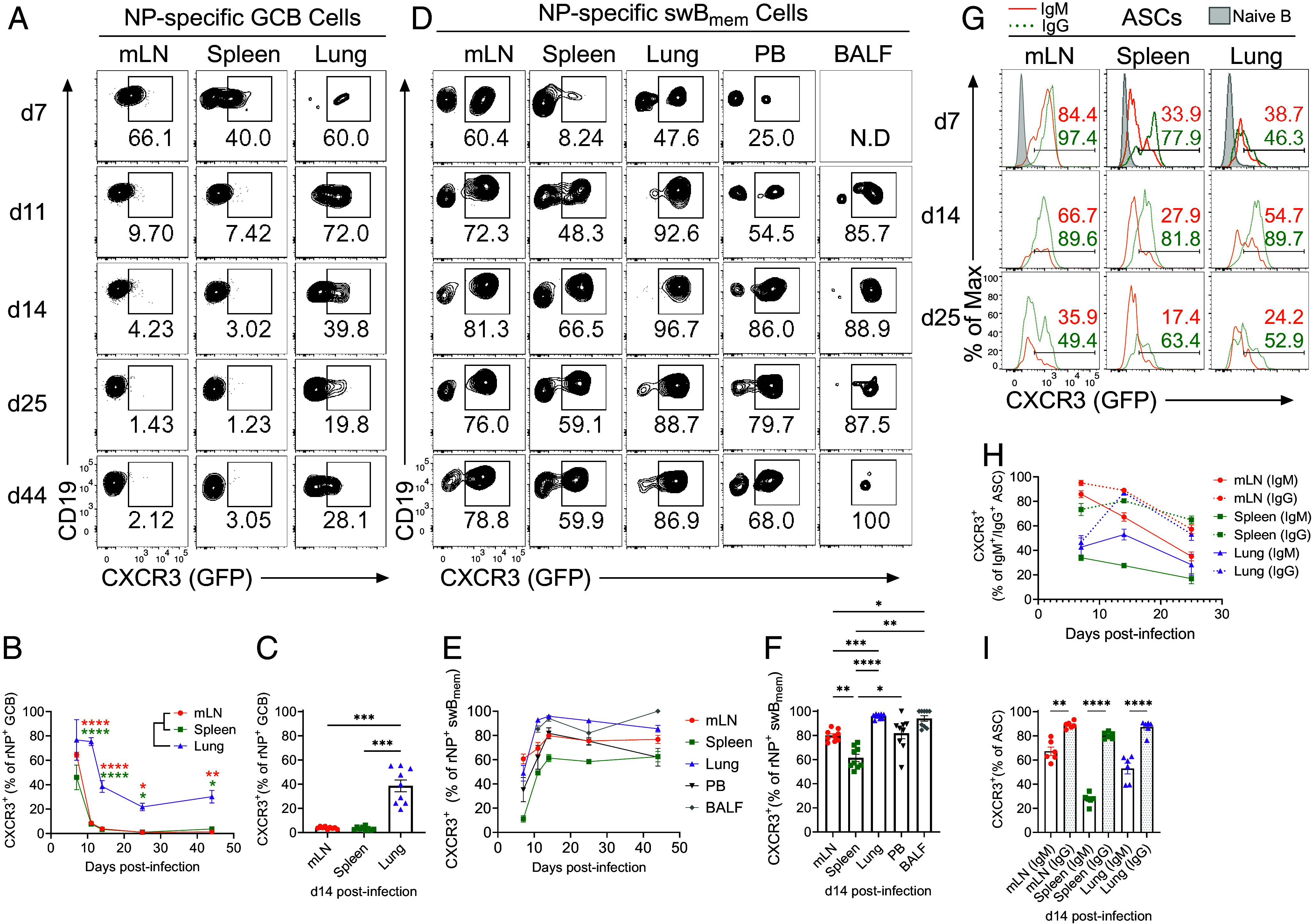
Ectopic pulmonary GCB cells and swB_mem_ cells are enriched for CXCR3 expression compared to their contemporaneous counterparts in SLOs following IAV infection. CIBER *Cxcr3*-reporter mice were infected with 10 TCID_50_ x31 IAV and the mLN, spleen, lung, PB and BALF were harvested on day 7, 11, 14, 25, and 44 postinfection for flow cytometric analysis. (*A*) Representative flow cytometry for longitudinal assessment of CXCR3-GFP expression in IAV NP-specific GCB cells. Flow plots are pregated on live, CD19^hi^, parenchymal, CD38^lo^GL7^+^rNP^+^ cells, as defined in *SI Appendix*, Fig. S1*A*. (*B* and *C*) Quantification of (*A*) for the time course assessed (*B*) and at d14 postinfection (*C*). (*D*) Representative flow cytometry for longitudinal assessment of CXCR3-GFP expression in IAV NP-specific swB_mem_ cells. Flow plots are pregated on live, TER119^−^ (PB, BALF), CD19^hi^, parenchymal (mLN, spleen, lung, BALF) or vascular (PB), IgD^−^IgM^−^CD38^hi^GL7^−^rNP^+^ (mLN, spleen, lung) or IgD^−^IgM^−^CD38^hi^rNP^+^ (PB, BALF) cells, as defined in *SI Appendix*, Fig. S1*A*. (*E* and *F*) Quantification of (*D*) for the time course assessed (*E*) and at d14 postinfection (*F*). (*G*) Representative flow cytometry for longitudinal assessment of CXCR3-GFP expression in IgM^+^ and IgG^+^ ASCs. Flow plots are pregated on live, parenchymal, CD3^−^CD4^−^CD8^−^NK1.1^−^IgD^−^CD11b^−^ and IgG^−^ (for IgM^+^ ASCs) or IgM^−^ (for IgG^+^ ASCs) cells, as depicted in *SI Appendix*, Fig. S1*B*. (*H* and *I*) Quantification of (*G*) for the time course assessed (*H*) and at d14 postinfection (*I*). Data presented as mean ± SEM. n = 4 to 9 mice/time point, pooled from 2 to 3 independent experiments. Data in (*B*) were analyzed by repeated measures two-way ANOVA with Bonferroni’s multiple comparison test and the statistic shown are for lung compared to both mLN and spleen. Data in (*C* and *F*) were analyzed by repeated measures one-way ANOVA with Bonferroni’s multiple comparisons test to compare all groups and data in (*I*) were analyzed by paired *t* tests to compare IgM and IgG ASCs within each organ. *P < 0.05, **P < 0.01, ***P < 0.001, ****P < 0.0001.

To provide an experimental framework for understanding CXCR3 expression kinetics among conventional and ectopic GCB cells following respiratory viral infection, we utilized a model of IAV infection in which ectopic pulmonary GCB cell responses develop in the first weeks postinfection, peak around day (d) 14 to 15 postinfection, and persist for several weeks thereafter (11–13, 33, 34, 50). In this model, CXCR3 expression kinetics in GCB cells, swB_mem_, and ASCs were assessed in SLOs, peripheral blood (PB), lungs, and bronchoalveolar lavage fluid (BALF) of IAV-infected CIBER *Cxcr3-*reporter mice (49). The use of CIBER *Cxcr3*-reporter mice was required to measure CXCR3 expression as the collagenase-mediated tissue digestion protocol utilized in this study for preparation of single-cell suspensions disrupts monoclonal antibody staining of cell-surface CXCR3. Representative gating strategies for identification of cell populations of interest in each anatomical location and their corresponding quantitation are provided in *SI Appendix*, Figs. S1 *A* and *B* and S2 *A*–*C*. CXCR3 reporter expression by B cells was negligible in naïve mice in all anatomical compartments assessed but was clearly apparent postinfection (*SI Appendix*, Fig. S3 *A* and *B*). Assessing IAV nucleoprotein (NP)-specific GCB cells across the time course in each anatomical compartment separately, CXCR3 expression was highest in the mediastinal LN (mLN) and spleen at d7 postinfection (mean in mLN: 64.7%, mean in spleen: 46.0%), and in the lung at d11 postinfection (mean in lung: 75.3%), when appreciable numbers of pulmonary GCB cells were detectable (Fig. 1 *A* and *B* and *SI Appendix*, Fig. S2*A*). Expression of CXCR3 among GCB cells waned over the ensuing weeks in all three compartments (Fig. 1 *A* and *B*). This pattern of CXCR3 expression was also apparent when total GCB cells in the mLN, spleen, and lung were analyzed (*SI Appendix*, Fig. S3 *C* and *D*). From d11 postinfection onward, CXCR3 expression was significantly elevated in ectopic GCB cells (both total and NP-specific) in lungs relative to their counterparts in SLOs (Fig. 1 *A*–*C* and *SI Appendix*, Fig. S3 *C* and *D*). Assessing the proportion of unswitched cells in CXCR3^+^ and CXCR3^−^ GCB cells revealed CXCR3^+^ GCB cells in SLOs are enriched for unswitched IgM relative to their CXCR3^−^ counterparts (*SI Appendix*, Fig. S3*E*). In contrast, lung CXCR3^−^ GCB cells are enriched for unswitched IgM^+^ cells compared to their CXCR3^+^ counterparts at d11 postinfection, when appreciable numbers of ectopic GCB cells are detectable (*SI Appendix*, Fig. S2*A*), before CXCR3^−^ and CXCR3^+^ ectopic GCB cells converge with respect to the frequency of unswitched B cells thereafter (*SI Appendix*, Fig. S3*E*). Overall, the results demonstrate CXCR3 is expressed by antigen-specific GCB cells in the mLN, spleen, and lung in response to IAV infection, and while CXCR3 is downmodulated by GCB cells as SLO- and lung-residing GCs mature between d7 and d25 postinfection, CXCR3 expression remains elevated on ectopic GCB cells in iBALT relative to GCB cells in SLOs.

Among swB_mem_ cells, CXCR3 was expressed by NP-specific and total swB_mem_ cells from all anatomical compartments assessed following IAV infection (Fig. 1 *D* and *E* and *SI Appendix*, Fig. S3 *F* and *G*). The utility of CXCR3 as a potential marker for tracking IAV-responding swB_mem_ cells was evidenced by the observation that a large proportion of NP-specific swB_mem_ cells from the mLN, spleen, lung, PB, and BALF were CXCR3^+^ from d14 postinfection onward (Fig. 1 *D* and *E*). Furthermore, CXCR3 expression was significantly enriched in NP-specific swB_mem_ cells compared to swB_mem_ cells that were not NP-reactive (*SI Appendix*, Fig. S3*H*), consistent with previous findings from Gregoire and colleagues demonstrating bona fide IAV antigen-specific MBCs are enriched for CXCR3 expression (51). Notably, CXCR3 expression was most prominent on NP-specific and total swB_mem_ cells isolated from effector sites in the lung and airways (Fig. 1 *D*–*F* and *SI Appendix*, Fig. S3 *F*–*I*).

Among ASCs, IgM^+^ and IgG^+^ ASCs present in the mLN, spleen, and lung following IAV infection also expressed abundant CXCR3 at the timepoints measured (Fig. 1 *G* and *H*). CXCR3 expression was generally higher on early ASCs at d7 postinfection in the mLN and spleen, and at d14 postinfection in the lung, while CXCR3 expression reduced by d25 postinfection (Fig. 1 *G* and *H*), phenocopying the temporal pattern of CXCR3 down modulation observed in GCB cells between d7 and d25 postinfection (Fig. 1 *A* and *B* and *SI Appendix*, Fig. S3 *C* and *D*). Additionally, CXCR3 expression was higher on IgG^+^ ASCs compared to IgM^+^ ASCs within the mLN, spleen, and lung at most timepoints assessed following IAV infection (Fig. 1 *G*–*I*). The high CXCR3 expression on lung ASCs suggests that CXCR3 may be involved in coordinating lung ASC generation and/or homing from SLOs in the context of IAV infection, consistent with its potential and established roles in driving ASC accumulation at inflamed sites in other settings (52–56). Together, these kinetic analyses demonstrate CXCR3 expression is a prominent feature of antigen-specific swB_mem_ cells and IgG^+^ ASCs following IAV infection.

### B Cell–Expressed CXCR3 Is Functional and CXCR3 Ligands Are Widely Expressed Following IAV Infection.

**Fig. 2.**
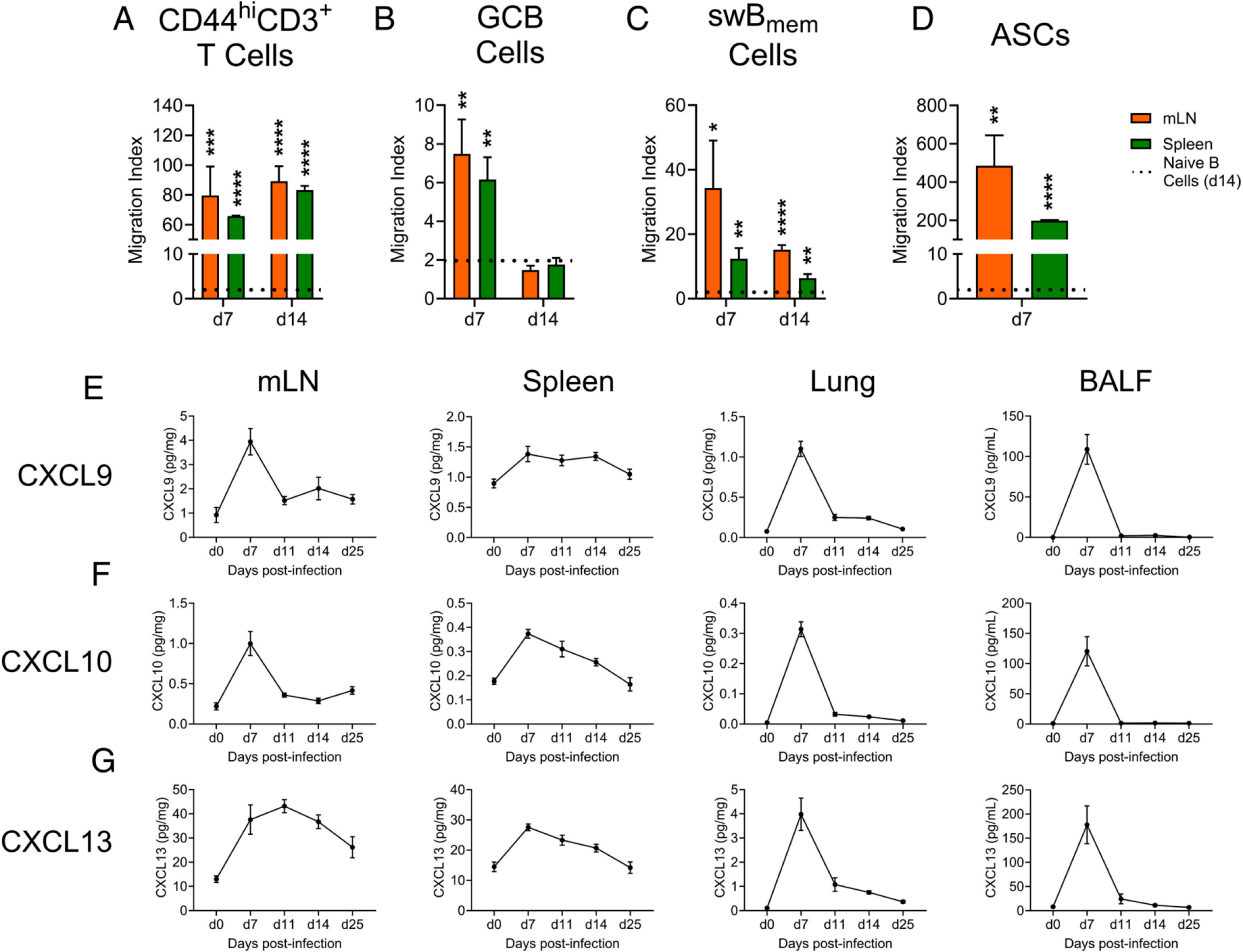
IAV infection-induced B cell–expressed CXCR3 is functional and CXCR3 ligands are upregulated in multiple anatomical compartments following IAV infection. WT mice were infected with 10 TCID_50_ x31 and the mLN and spleen were harvested on day 7 and 14 postinfection. Single-cell suspensions prepared from the mLN and spleen were subjected to ex vivo transwell chemotaxis assays to assess cell migration to CXCL11 (500 ng/mL). (*A*–*D*) Migration index for the indicated cell populations in the mLN and spleen at day 7 and 14 postinfection. The dashed line in graphs indicates the migration index of naïve B cells at d14 postinfection. (*E*–*G*) CIBER mice were infected with 10 TCID_50_ x31 IAV and the mLN, spleen, lung, and BALF were harvested on day 7, 11, 14, and 25 postinfection. Supernatants were prepared from digested tissues and BALF washes and analyzed by multiplex flow cytometry assays to assess the levels of (*E*) CXCL9, (*F*) CXCL10 and (*G*) CXCL13. Data presented as mean ± SEM. (*A*–*D*) n = 3 biological replicates/organ/timepoint, representative of two similar experiments. (*E*–*G*) n = 4 to 6 biological replicates/anatomical compartment/timepoint, pooled from two independent experiments. Data in (*A*–*D*) were analyzed by the unpaired *t* test to compare each cell population in each organ at each timepoint to naïve B cells at d14 postinfection. *P < 0.05, **P < 0.01, ***P < 0.001, ****P < 0.0001.

To determine whether B cell expression of CXCR3 functionally mediates chemotaxis, bulk single-cell suspensions prepared from whole mLNs or spleens of IAV-infected mice at d7 and d14 postinfection were subjected to ex vivo transwell chemotaxis assays to assess cell migration following CXCR3 ligation. As a control, activated CD44^hi^CD3^+^ T cells migrated strongly toward CXCL11 (Fig. 2*A*). Moreover, GCB cells, swB_mem_ cells, and ASCs migrated toward CXCL11 to varying degrees (Fig. 2 *B*–*D*), with the pattern of sensitivity to CXCL11 largely reflecting the cell type–specific and temporal differences in CXCR3 expression observed on responding B cells (Fig. 1 *A*–*I* and *SI Appendix*, Fig. S3 *C*, *D*, *F*, and *G*). Together, these results demonstrate that responding activated B cell populations in IAV-infected mice functionally express CXCR3.

Production of CXCR3 ligands expressed in C57Bl/6 mice (CXCL9 and CXCL10) was also assessed in the relevant anatomical compartments following IAV infection (Fig. 2 *E* and *F*). CXCL11 was not assessed as it is not expressed in mice on a C57Bl/6 background (57). CXCL9 and CXCL10 protein increased from basal levels in the lung and BALF, as well as the mLN and spleen (Fig. 2 *E* and *F*), peaking at d7 postinfection in all compartments assessed, while remaining elevated at d11 and d14 postinfection in the mLN, spleen, and lung (Fig. 2 *E* and *F*). Additionally, CXCL13 levels, which serve as a surrogate for iBALT formation (11), were increased in the lung following IAV infection and remained elevated above baseline until at least d25 postinfection (Fig. 2*G*). Thus, ligands for CXCR3, which is functional on responding B cell populations, are induced in SLOs and in the lung following intranasal IAV infection.

### CXCR3 in B Cells Drives Efficient Generation of Ectopic GCB Cells in the Lung Following IAV Infection.

**Fig. 3.**
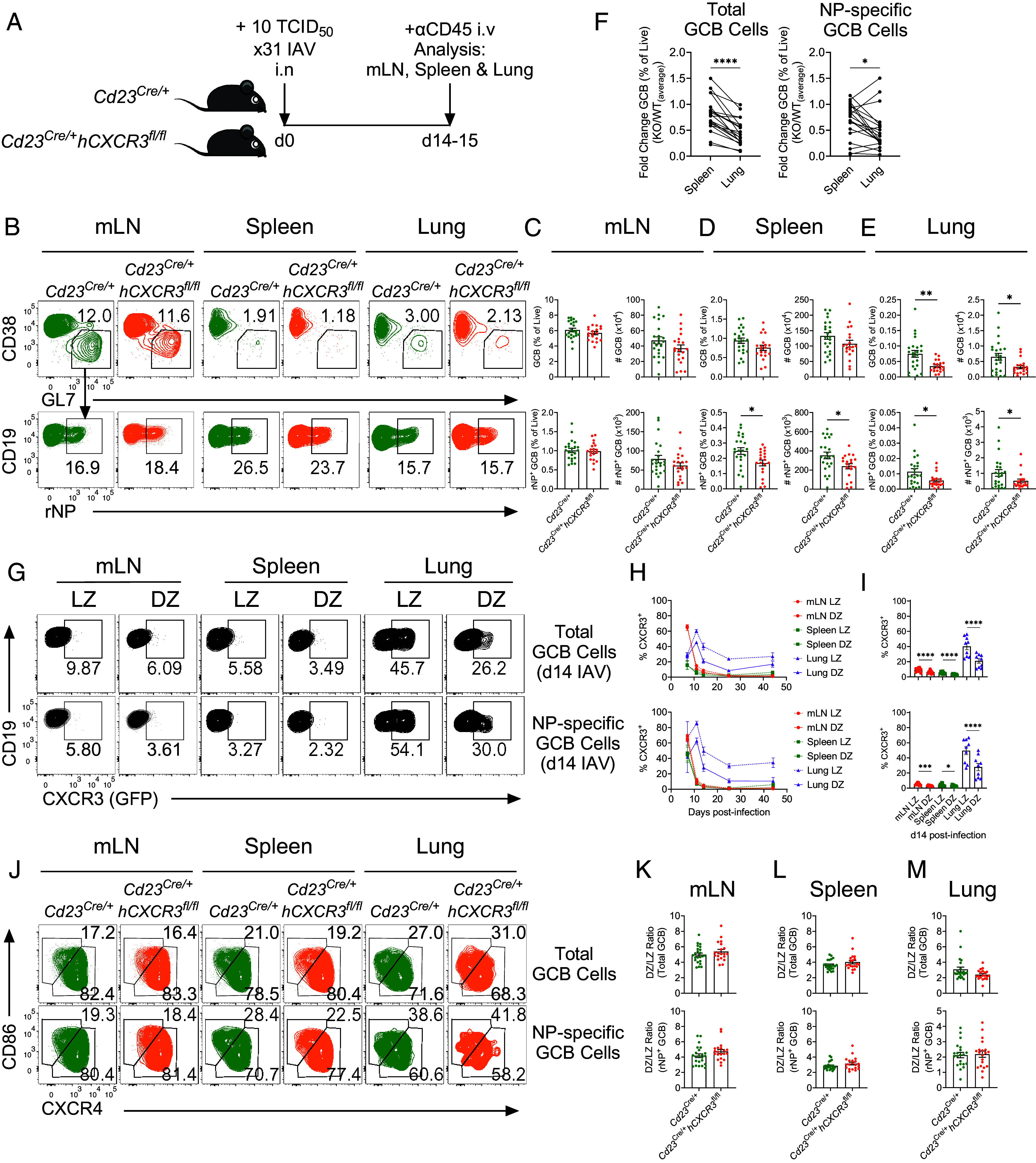
Lack of CXCR3 expression in B cells inhibits generation of ectopic GCB cells in the lung following IAV infection. (*A*–*M*) *Cd23^Cre/+^* and *Cd23^Cre/+^hCXCR3^fl/fl^* mice (*A*–*F* and *J*–*M*) or CIBER mice (*G*–*I*) were infected with 10 TCID_50_ x31 IAV and the mLN, spleen, and lung were harvested on day 14 to 15 postinfection for flow cytometric analysis. (*A*) Experimental schematic for experiments in *Cd23^Cre/+^* and *Cd23^Cre/+^hCXCR3^fl/fl^* mice. (*B*) Representative flow cytometry for identification of total (*Top*) and IAV NP-specific (*Bottom*) GCB cells in the mLN, spleen, and lung. Flow plots for total GCB cells (*Top*) are pregated on live, B220^+^CD19^+^CD45-IV^−^ cells. (*C*–*E*) Quantification of (*B*) by percentage of total live cells (*Left*) and number (*Right*) for total (*Top*) and IAV NP-specific (*Bottom*) GCB cells in the (*C*) mLN, (*D*) spleen and (*E*) lung. (*F*) Fold change in total (*Left*) and IAV NP-specific (*Right*) GCB cell frequencies in the spleen and lung of *Cd23^Cre/+^hCXCR3^fl/fl^* (KO) mice normalized to their average frequencies in *Cd23^Cre/+^* (WT) mice for each anatomical location. Normalized GCB cell frequencies in the spleen and lung are connected for each mouse. (*G*) Representative flow cytometry assessing CXCR3-GFP expression in total (*Top*) and IAV NP-specific (*Bottom*) LZ and DZ GCB cells from CIBER mice. (*H* and *I*) Quantification of (*G*) for the time course assessed (*H*) and at d14 postinfection (*I*). (*J*) Representative flow cytometry for identification of LZ and DZ compartments within total (*Top*) and IAV NP-specific (*Bottom*) GCB cells in the mLN, spleen, and lung, as defined in Fig. 3*B*. (*K*–*M*) DZ/LZ ratio in total (*Top*) and IAV NP-specific (*Bottom*) GCB cells, depicted in (*J*), from the (*K*) mLN, (*L*) spleen and (*M*) lung. Data in (*C*–*E*, *H*, *I*, and *K*–*M*) are presented as mean ± SEM. (*B*–*F* and *J*–*M*) n = 20 to 23 mice/group, pooled from eight independent experiments. (*G*–*I*) n = 5 to 9 mice/time point, pooled from 2 to 3 independent experiments. Data in (*C*–*E*) were analyzed by unpaired *t* tests. Data in (*F*) were analyzed by paired *t* tests. Data in (*I*) were analyzed by paired *t* tests to compare LZ and DZ GCB cells within each organ. *P < 0.05, **P < 0.01, ***P < 0.001, ****P < 0.0001.

To address the function of CXCR3 in B cells during IAV infection, we crossed mice expressing Cre under control of the promoter of the follicular B cell–specific gene *Cd23* (*Cd23^Cre/+^*), with mice containing a floxed human *CXCR3* gene (*hCXCR3^fl/fl^*), thereby generating B cell–specific CXCR3-deficient mice (*Cd23^Cre/+^hCXCR3^fl/fl^*), which lack CXCR3 expression on B cells (*SI Appendix*, Fig. S4 *A* and *B*). Importantly, with relevance to the mouse models employed here, murine and human CXCR3 are both equivalently activated by murine CXCR3 ligands (58, 59). We infected these mice with IAV and assessed GCB cell responses in SLOs and lung iBALT on d14-15 postinfection using flow cytometry (Fig. 3*A*). Analysis of GCB cells in SLOs from *Cd23^Cre/+^* and *Cd23^Cre/+^hCXCR3^fl/fl^* mice revealed that total and NP-specific GCB cell responses were unaltered in the mLN (Fig. 3 *B* and *C*). In the spleen, total GC induction was unaltered between groups (Fig. 3 *B* and *D*). However, splenic IAV NP-specific GCB cells were significantly reduced when B cells lacked CXCR3 expression (Fig. 3 *B* and *D*). Consistent with a role for CXCR3 in the generation of ectopic GCB cell responses, both total and NP-specific GCB cell responses were impaired in the lung of B cell specific CXCR3-deficient mice relative to control mice (Fig. 3 *B* and *E*). Notably, the magnitude of the defect in GCB cell responses in the absence of B cell–expressed CXCR3 was markedly greater in the lung compared to the spleen (Fig. 3*F*). Analysis of IgM positivity among lung GCB cells revealed a small yet significant increase in the frequency of IgM^+^ NP-specific GCB cells, while IgM positivity in SLO GCs was unchanged, suggesting lack of CXCR3 expression in B cells compromises class-switching in ectopic GCB cells in the lung (*SI Appendix*, Fig. S5 *A* and *B*). Kinetic analysis of CXCR3 expression in GCB cells in *CIBER* mice revealed elevated CXCR3 expression in light zone (LZ) cells compared to their dark zone (DZ) counterparts (Fig. 3 *G*–*I*). To assess whether the observed defects in spleen and lung GCB cell responses between *Cd23^Cre/+^* and *Cd23^Cre/+^hCXCR3^fl/fl^* mice were due to CXCR3-mediated regulation of GC polarization, GC DZ/LZ ratios were evaluated by flow cytometry. Despite a report indicating that CXCR3 promotes access of GCB cells to the DZ in the spleen during malaria infection (41), the DZ/LZ ratios within GCs in the mLN, spleen, and lung at d14-15 postinfection with IAV were unaffected by loss of CXCR3 in B cells (Fig. 3 *J*–*M*). Thus, splenic and pulmonary GCB cell responses are compromised following IAV infection when B cells lack CXCR3, but this is not driven by defects in GC polarization. Overall, these results demonstrate that CXCR3 is required by B cells for efficient generation of GC responses in lung iBALT following IAV infection.

In parallel to analyses of the GCB cell response, swB_mem_ cells were also evaluated at d14-15 postinfection with IAV (*SI Appendix*, Fig. S6 *A*–*D*). At this timepoint, prior reports have indicated that swB_mem_ in the lung are mainly BRM cells, originating from GC-dependent precursors that likely home to the lung from the mLN in the first few weeks postinfection (34, 36). Notably, CXCR3 is highly expressed by IAV-responding swB_mem_ cells in the PB, lung, and airways (Fig. 1 *D*–*F* and *SI Appendix*, Fig. S3 *F*–*H*), and CXCR3 is known to promote lung-homing of T cells and NK cells during IAV infection (24, 25, 60, 61). Combined, these findings raised the possibility that CXCR3 may coordinate lung-homing of swB_mem_ cells from SLOs and subsequent establishment of the local BRM cell pool. While the results demonstrate that the generation of swB_mem_ cells in SLOs is unaffected by loss of CXCR3 expression in B cells (*SI Appendix*, Fig. S6 *A*–*C*), the accumulation of swB_mem_ cells in the lung was also unaltered by CXCR3 deletion (*SI Appendix*, Fig. S6 *A* and *D*). Further, there were no differences in the frequency or number of total or NP-specific swB_mem_ cells in the PB and BALF (*SI Appendix*, Fig. S6 *E*–*G*). Thus, although CXCR3 is highly expressed by circulating and lung-infiltrating IAV-specific swB_mem_ cells at d14-15 postinfection, it is dispensable for their generation in SLOs and their accumulation in the lung at the peak of the response following primary IAV infection, although previous findings demonstrate CXCR3 is required for establishment of a subset of BRM cells producing IgA at 5 wk postinfection with IAV (47).

**Fig. 4.**
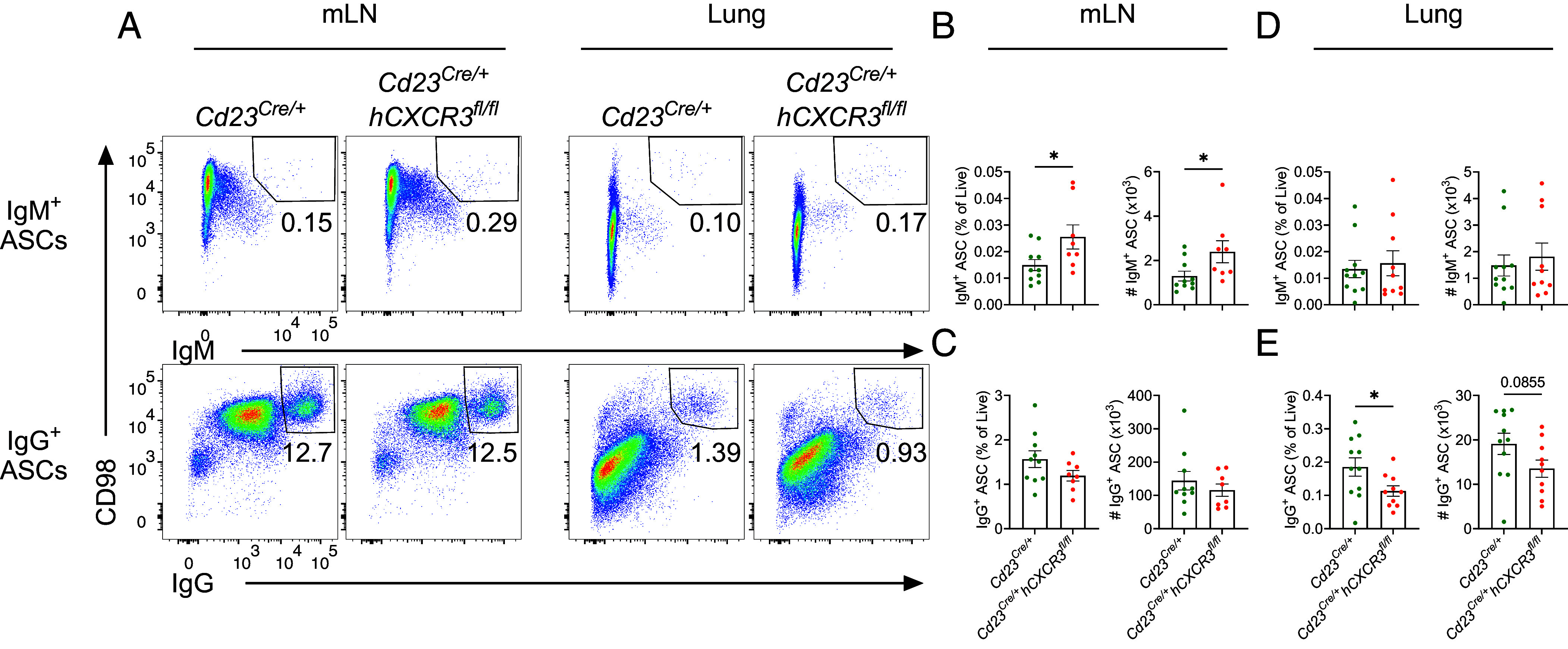
Lack of CXCR3 expression in B cells impairs the accumulation of IgG-secreting ASCs in the lung following IAV infection. *Cd23^Cre/+^* and *Cd23^Cre/+^hCXCR3^fl/fl^* mice were infected with 10 TCID_50_ x31 IAV and the mLN and lung were harvested on day 14 to 15 postinfection for flow cytometric analysis. (*A*) Representative flow cytometry for identification of IgM^+^ ASCs (*Top*) and IgG^+^ ASCs (*Bottom*) in the mLN and lung. Flow plots are pregated on live, parenchymal (αCD45-IV^−^), CD3^−^CD4^−^CD8^−^NK1.1^−^IgD^−^CD11b^−^ and IgG^−^ (for IgM^+^ ASCs) or IgM^−^ (for IgG^+^ ASCs) cells. (*B*–*E*) Quantification of (*A*) in terms of percentage of total live cells (*Left*) and number (*Right*) for IgM^+^ ASCs (*B* and *D*) and IgG^+^ ASCs (*C* and *E*) in the (*B* and *C*) mLN and (*D* and *E*) lung. Data are presented as mean ± SEM. n = 8 to 11 mice/group, pooled from four independent experiments. Data in (*B*–*E*) were analyzed by unpaired *t* tests. *P < 0.05.

Oh et al. have previously shown CXCR3 is required for establishment of protective IgA-producing ASCs in the lung following IAV infection (47). Furthermore, previous single-cell BCR sequencing analyses have identified the presence of clonal overlap between IAV-responding B cell populations in the mLN and lung with ASCs in the lung (47, 62), suggesting pulmonary ASCs are derived from both systemic and local sources. To determine whether CXCR3 expression in B cells is required for generation of other Ig-producing ASC responses in the lung, IgM^+^ and IgG^+^ ASCs in *Cd23^Cre/+^* and *Cd23^Cre/+^hCXCR3^fl/fl^* mice were evaluated at the peak of the response on d14-15 postinfection with IAV. Although scarce, IgM^+^ ASCs in the mLN were increased when B cells could not express CXCR3, while the more prolific IgG^+^ ASC response in the mLN did not rely on CXCR3 expression in B cells (Fig. 4 *A*–*C*). Interestingly, accumulation of IgG^+^ ASCs in the lung was impaired when B cells lacked CXCR3, possibly reflecting the concomitant defect in class-switching observed in CXCR3-deficient lung GCs (*SI Appendix*, Fig. S5*B*), while the frequencies of IgM^+^ ASCs in the lung were unchanged (Fig. 4 *A*, *D*, and *E*). Thus, the results indicate that CXCR3 is required in B cells to drive accumulation of IgG^+^ ASCs in the lung at the peak of the response to IAV infection, reflecting the previously identified role of CXCR3 in establishment of IgA^+^ ASCs in the lung (47).

### Loss of CXCR3 Expression in B Cells Does Not Affect Early or Late-Phase B Cell Responses to Primary IAV Infection.

BCR sequencing and clonal overlap studies indicate ectopic pulmonary GCB cell responses in iBALT following IAV infection appear to be mainly derived from priming of naïve B cells in the lung (13, 62, 63). However, there is a small degree of clonal overlap between responding B cell populations in SLOs and ectopic GCB cells in the lungs, suggesting SLO-primed precursors that migrate to the lungs also contribute to ectopic pulmonary GCs to a minor extent. Therefore, to evaluate the possibility that defective generation of ectopic pulmonary GCB cell responses on d14-15 postinfection with IAV in the context of B cell–specific CXCR3 deficiency was related to impaired early induction of B cell responses in SLOs, *Cd23^Cre/+^* and *Cd23^Cre/+^hCXCR3^fl/fl^* mice were infected with IAV and the mLN and spleen were assessed on d7 postinfection by flow cytometry. There were no significant differences in the early generation of total and NP-specific GCB cells (*SI Appendix*, Fig. S6 *H*–*J*) or ASCs (*SI Appendix*, Fig. S6 *K*–*M*) in the mLN and spleen from *Cd23^Cre/+^* and *Cd23^Cre/+^hCXCR3^fl/fl^* mice. Thus, although CXCR3 is highly expressed by early responding B cell populations in SLOs, it is not required for initiation or priming of the B cell response in the mLN or spleen following IAV infection.

A hallmark of the B cell response to primary IAV infection is the generation of a long-lived MBC pool that provides protection against reinfection (12, 33, 34, 48, 51, 63). Notably, tissue-resident MBCs are established in IAV-infected lungs and this pool of memory cells is characterized by elevated frequencies of cross-reactive MBCs derived from hypermutated precursors inhabiting persistent ectopic GCs within iBALT (12, 34). Here, we found persistent GCB cells in the lung express elevated levels of CXCR3 compared to their counterparts in SLOs (Fig. 1 *A* and *B*) and CXCR3 is highly expressed by bona fide, IAV-specific, swB_mem_ cells (Fig. 1 *D*–*F*). However, the role of CXCR3 in persistent GCB cell responses and MBC responses during the late-phase of the response to IAV infection is incompletely understood. Thus, *Cd23^Cre/+^* and *Cd23^Cre/+^hCXCR3^fl/fl^* mice were infected with IAV and the mLN, spleen, and lung were harvested on d42 postinfection for flow cytometric analysis. There were no differences between groups with respect to the frequency and number of total or NP-specific GCB cells or swB_mem_ cells in the mLN, spleen, and lung in the late-phase of the response (*SI Appendix*, Fig. S6 *N*–*U*). Taken together, while lack of CXCR3 expression in B cells leads to impaired generation of pulmonary GCB cell responses in iBALT at the peak of the response to IAV, B cell–expressed CXCR3 is not required for initiation of B cell responses in SLOs or late-phase GCB cell and swB_mem_ cell responses following IAV infection.

### CXCR3 Is Required by B Cells to Ensure Competitive Fitness for Participation in Ectopic GCs Following IAV Infection.

**Fig. 5.**
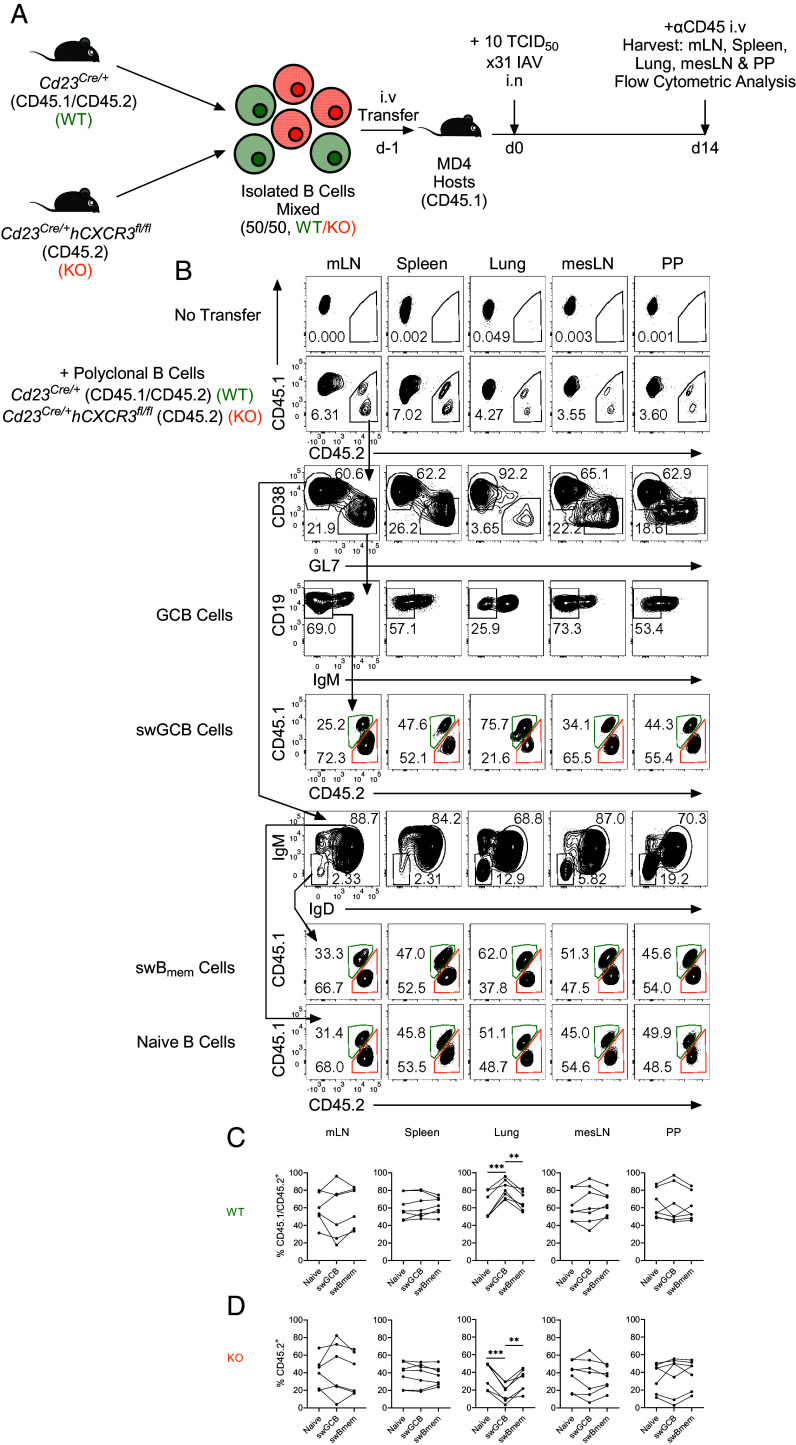
Lack of CXCR3 expression in B cells restricts their competitive fitness for participation in ectopic pulmonary GC responses following IAV infection. B cells were isolated from the spleens of naïve *Cd23^Cre/+^* (CD45.1/CD45.2) and *Cd23^Cre/+^hCXCR3^fl/fl^* (CD45.2) mice, mixed, and transferred intravenously to MD4 (CD45.1) hosts at doses of 15 to 30 × 10^6^ total B cells. MD4 host mice were then intranasally infected with 10 TCID_50_ x31 IAV the following day. On day 14 postinfection, the mLN, spleen, lung, mesLN, and PP were harvested from MD4 host mice, after i.v-labeling, for flow cytometric analysis. (*A*) A schematic of the experiment. (*B*) Representative flow cytometry for identification of donor-derived *Cd23^Cre/+^* (WT) and *Cd23^Cre/+^hCXCR3^fl/fl^* (KO) swGCB cells, swB_mem_ cells, and naïve B cells. Flow plots in rows 1 and 2 are pregated on live, B220^+^CD19^+^CD45-IV^−^ cells. (*C* and *D*) The proportions of CD45.1/CD45.2^+^ *Cd23^Cre/+^* (WT) (*C*) or CD45.2^+^ *Cd23^Cre/+^hCXCR3^fl/fl^* (KO) (*B*) cells among donor-derived naïve B cells, swGCB cells, and swB_mem_ cells in the mLN, spleen, lung, mesLN, and PP of MD4 hosts. n = 6 to 7 mice/organ, pooled from three independent experiments. Data in (*C* and *D*) were analyzed by paired *t* tests to compare naïve B cells with swGCB cells or swGCB cells with swB_mem_ cells. **P < 0.01, ***P < 0.001.

The observation that efficient generation of IAV-induced pulmonary GCB cells required B cell–expressed CXCR3 (Fig. 3 *A*–*F*) prompted us next to question the competitive fitness of CXCR3-deficient B cells. To test this, a cotransfer model was established utilizing adoptive transfer of congenically marked polyclonal B cells, purified from naïve *Cd23^Cre/+^* and *Cd23^Cre/+^hCXCR3^fl/fl^* mice, into BCR transgenic MD4 hosts that were subsequently infected with IAV. MD4 mice were used as host recipient mice as >95% of their endogenous B cells express a transgenic BCR recognizing HEL (64, 65), an irrelevant BCR specificity in the context of IAV infection, thereby restricting responses to the adoptively transferred polyclonal B cells following IAV infection, reflecting previous studies using MD4 mice as hosts in this context (66, 67). Congenically distinct polyclonal B cells purified from naïve *Cd23^Cre/+^* and *Cd23^Cre/+^hCXCR3^fl/fl^* mice were mixed in equal parts and adoptively transferred into MD4 (CD45.1^+^) hosts that were subsequently infected with IAV. On d14 postinfection, the mLN, spleen, and lung were subjected to flow cytometric analysis to assess the contribution of CD45.1^+^/CD45.2^+^ *Cd23^Cre/+^* (WT) or CD45.2^+^ *Cd23^Cre/+^hCXCR3^fl/fl^* (KO) B cells to the donor-derived naïve B cell, class-switched GCB (swGCB) cell, total GCB cell, and swB_mem_ cell compartments (Fig. 5*A*). The results revealed that *Cxcr3*-deficient B cells are significantly underrepresented in the pulmonary donor-derived swGCB cell compartment when compared to their contemporaneous corresponding frequencies in the naïve B cell compartment (Fig. 5 *B*–*D*), a result reflected in the analysis of WT and KO B cell frequencies among lung donor-derived total GCB cells (*SI Appendix*, Fig. S7 *A* and *B*), while WT and KO B cells contributed equally to swGCB cell and total GCB cell responses in the mLN and spleen (Fig. 5 *B*–*D* and *SI Appendix*, Fig. S7 *A* and *B*). This indicates that B cell–expressed CXCR3 is required to ensure competitive fitness of responding B cells in ectopic GCs within lung iBALT. Interestingly, while there was no apparent role for CXCR3 in modulating lung infiltration of swB_mem_ cells (*SI Appendix*, Fig. S7 *C* and *D*), KO B cells were significantly overrepresented in donor-derived swB_mem_ cells in the lung when compared to their frequencies in contemporaneous swGCB cells, their potential precursors, suggesting lack of CXCR3 leads to increased output of MBCs from pulmonary GCs. However, this finding likely stems from the phenomenon that a majority of swB_mem_ cells in the lung at this timepoint originate from SLO-residing GCs (34, 36), in which B cell participation appears to be CXCR3-independent (Fig. 5 *B*–*D* and *SI Appendix*, Fig. S7 *A* and *B*). Combined, the results demonstrate that CXCR3 is required by B cells to ensure competitive fitness in ectopic GC reactions in the lung iBALT following IAV infection. This outcome supports our findings obtained from assessing IAV-infected *Cd23^Cre/+^* and *Cd23^Cre/+^hCXCR3^fl/fl^* mice as individual hosts (Fig. 3 *A*–*F*). Moreover, the CXCR3-dependent competitive advantages in GC participation were restricted to IAV-induced GCB cell responses in the lung, as there was no apparent role for B cell–expressed CXCR3 in regulation of GCB cell responses, likely against gut microflora (66), that dominate the chronic GC reactions in the mesenteric LN (mesLN) and Peyer’s Patches (PP) (Fig. 5 *B*–*D*).

## Discussion

Maturation of B cell responses require chemoattractant receptors to be dynamically and spatiotemporally regulated, guiding the B cell into distinct microenvironmental niches in a timely manner to receive their proliferative and differentiation cues. Thus, from a clinical perspective, gaining a comprehensive understanding of the suite of chemoattractant receptors underpinning successful B cell responses in diverse settings is of paramount importance. Here, we demonstrate that CXCR3 is an important component of the B cell response to primary IAV infection. By tracking CXCR3 expression using CIBER *Cxcr3*-reporter mice, we reveal that CXCR3 is prominently expressed by multiple responding B cell populations, being significantly enriched on ectopic GCB cells and swB_mem_ cells at the site of infection in lung iBALT relative to their counterparts in SLOs. Investigations using B cell–specific CXCR3-deficient (*Cd23^Cre/+^hCXCR3^fl/fl^*) mice demonstrated CXCR3 in B cells drives efficient generation of pulmonary GCB cell responses in iBALT following IAV infection in both isolated and competitive settings, while the initiation of the B cell response in SLOs and the generation of late-phase systemic and local swB_mem_ cell responses remained largely intact in the absence of B cell–expressed CXCR3.

Whereas the chemoattractant receptors responsible for coordinating GCB cell biology in SLOs are relatively well-characterized (5), the chemotactic requirements underpinning efficient ectopic GC reactions in ELTs such as iBALT are poorly understood. Furthermore, whether conventional and ectopic GCs bear differential chemokine receptor dependencies is unknown. The results from this study elucidate CXCR3 as a B cell–intrinsic requirement for generation of pulmonary GCB cell responses in iBALT, detailing a B cell–expressed chemokine receptor selectively involved in facilitating ectopic GC reactions. While CXCR3 deficiency in B cells resulted in a profound defect in the generation of pulmonary GCB cells at the peak of the response to IAV on d14-15 postinfection, the cellularity of the late-phase pulmonary GC reaction was unaffected by lack of CXCR3 in B cells. These findings suggest B cell–expressed CXCR3 supports efficient initiation and/or amplification of pulmonary GCB cell responses, while it appears to be dispensable for their prolonged maintenance, despite elevated CXCR3 expression among lung B cell populations compared to their contemporaneous counterparts in SLOs.

While CXCR3 deficiency in B cells led to defective generation of pulmonary GCB cell responses in iBALT, the CXCR3-dependent mechanisms underpinning this phenotype remain unclear. In contrast to the high degree of clonal overlap between IAV-induced SLO GCB cells and lung BRM cells identified by single-cell BCR sequencing, there is only a minor level of shared clonotypes between pulmonary GCB cells in iBALT and contemporaneous responding B cell populations in SLOs, indicating a majority of clones in pulmonary GCs are specific to the lung (13, 62, 63). Thus, current knowledge suggests IAV-induced pulmonary GCB cell responses in iBALT are derived mainly from in situ primed naïve B cells, which are recruited to iBALT from circulation via the CXCR5–CXCL13 axis (11), and to a lesser extent, SLO-derived precursors. Considering this, it is conceivable that CXCR3 may support generation of pulmonary GCB cell responses via several avenues. For instance, B cell–expressed CXCR3 may regulate migratory events required for B cell responses within the unique iBALT microenvironment of the IAV-infected lung. Indeed, pre-GC B cells in the lung at early timepoints express CXCR3 (*SI Appendix*, Fig. S8), and our results indicate that CXCR3 ligands CXCL9 and CXCL10 are abundant in the lung early after IAV infection, supporting a possible role for CXCR3 in coordinating migratory events involved in priming of naïve B cells in iBALT or reactivation of ectopic GC-seeding precursors in the infected lung. CXCR3 may regulate these processes via several mechanisms, including directing proper interstitial localization of responding B cells in the lung and potentially promoting their access to antigen within GCs, although DZ/LZ GCB cell phenotypes were unaltered in this study. Further, CXCR3 may aid B cell acquisition of help from accessory cells such as T follicular helper cells in iBALT, reflecting the functions of CXCR3 in responding T cells (16–21, 28–32, 68–72). Additionally, while further investigations are required to elucidate the phenotype of migratory SLO-primed ectopic GC-seeding precursors, CXCR3 is known to facilitate trafficking of responding T cells and NK cells to the inflamed lung (24, 25, 60), inviting the possibility that CXCR3 may facilitate lung-homing of ectopic GC-seeding precursors to the IAV-infected lung, thereby contributing to the induction of pulmonary GCB cell responses. However, this is less likely to underlie the defect in generation of pulmonary GCB cell responses at the peak of the response on d14-15 postinfection with IAV, as clonal overlap studies suggest minimal sharing of clonotypes between SLO-responding B cells and lung GCs, and observations in the current study demonstrate swB_mem_ cells in the lung, which are primarily derived from SLO-originating, GC-dependent precursors in the mLN that migrate to the lungs (34, 51, 62, 63), do not rely on CXCR3 for accumulation in the lungs.

Further investigations are necessary to gain insight into the potential mechanisms underlying the requirement for B cell–expressed CXCR3 in efficient generation of ectopic pulmonary GCB cell responses in iBALT. Detailed in situ imaging studies in IAV infected lungs assessing localization of responding B cells, including CXCR3^+^ and CXCR3^−^ GCB cells, relative to both the position of T follicular helper (T_FH_) cells in iBALT, a critical component of pulmonary GC reactions (11), and the spatiotemporal distribution of CXCR3 ligands, could provide important insights into the mode of function of B cell–expressed CXCR3 in generation of lung GCB cell responses following primary respiratory viral infection. Furthermore, RNA sequencing analyses comparing the transcriptomes of CXCR3^+^/CXCR3^−^ or CXCR3 WT/KO GCB cells to assess for gene expression signatures of helped cells could inform whether CXCR3 in ectopic GCB cells promotes access to GC-supportive T cell help (73).

The generation of systemic and localized MBCs is also crucial for protective humoral immunity against microbial infections, such as IAV (74–77). Our spatiotemporal analysis of the B cell response following IAV infection revealed that IAV-specific swB_mem_ cells are broadly dispersed across multiple anatomical compartments in mice, including the mLN, spleen, PB, lung, and airways, consistent with the results reported by others assessing IAV-specific MBC responses in mice and humans (12, 33, 34, 48, 51, 62, 63, 78–80). Interestingly, swB_mem_ cells were observed in the BALF, a majority of which expressed CXCR3, and these cells may serve as strategically positioned early responders against secondary challenge, similar to the role of tissue-resident memory T cells in the airways in the context of IAV infection (81). IAV-specific swB_mem_ cells from various anatomical compartments were found to express high levels of CXCR3, in line with other reports (33, 34, 79), especially IAV-specific swB_mem_ cells at the site of infection in the lung iBALT, which are now recognized as BRM cells (34, 75). In vivo temporal disruptions in GC activity and lymphocyte egress from SLOs, in combination with BCR clonal overlap analyses, indicate BRM cells are likely derived from GC-dependent precursors in SLOs, primarily the lung draining mLN, which seed the lung during the first 3 wk postinfection (34, 51, 62, 63), although local pulmonary GC reactions provide important contributions to the IAV-specific MBC pool in the lung (12). “Prememory precursor” (pre-MEM) cells found in mLN GCs exhibit a LZ phenotype, express an MBC-related transcriptome, display high levels of CXCR3 and depend on T_FH_ cell-derived IFNγ (36). Indeed, IFNγ deficiency in T_FH_ cells or disruption of IFNγ signaling in B cells, through ablating IFNγR1, STAT1, or T-bet, specifically compromises the generation of CXCR3^+^ pre-MEM cells in mLN GCs and BRM cells in the lung following IAV infection (36). Given the prominent expression of CXCR3 across IAV-induced MBCs and the intimate association between CXCR3 and BRM cell biology, it is somewhat surprising that both systemic and localized MBC responses appear largely intact following IAV infection in the absence of B cell–expressed CXCR3, as observed in the present study. However, this is in line with other reports examining the role of CXCR3 in the generation of lung MBCs following IAV infection using competitive and noncompetitive mixed bone marrow chimeras (51, 63). Thus, CXCR3 is largely dispensable for the generation of systemic and localized MBCs following IAV infection, although it should be noted that CXCR3 is specifically required for establishment of IgA-producing BRM cells in the lung following IAV infection (47).

Recent investigations have revealed important roles for CXCR3 in secondary B cell responses to viral infections, including IAV. CXCR3 directs trafficking of migrant MBCs to the inflamed female reproductive tract upon secondary challenge with HSV, facilitating rapid induction of local protective antiviral antibody responses (82). Additionally, CXCR3 is required by B cells for optimal protection against secondary infection with a homologous strain of IAV (47). In line with this finding, CXCR3 promotes rapid relocation of BRM cells to infected alveoli upon secondary challenge with IAV, potentiating rapid, localized, protective antibody responses (48). Thus, in addition to the requirement for B cell expressed CXCR3 for efficient generation of primary ectopic GCB cell responses in iBALT identified in the present study, CXCR3 is emerging as a key functional regulator of MBC responses, reflecting the immunological importance of CXCR3 in reactivation and elicitation of memory T cell responses following secondary challenges (31, 32, 72). However, further work is required to assess the extent of CXCR3-mediated regulation of local and systemic secondary B cell responses in the context of IAV infection and other challenges, and to specifically evaluate whether CXCR3 is required by B cells for generation of secondary GC responses in the lung and SLOs after secondary infection with IAV.

In the present study, lack of CXCR3 in B cells led to a reduction in the generation of lung IgG-producing ASCs following IAV infection, while IgG-producing ASC responses in the mLN were unaffected. This suggests that CXCR3 is selectively involved in establishing local class-switched ASC responses following primary infection with IAV, a result supported by a recent study demonstrating successful establishment of IgA-producing lung ASCs following IAV infection requires B cell–expressed CXCR3 (47). However, the mechanisms underlying the CXCR3-dependent nature of pulmonary class-switched ASC responses in the context of IAV infection remain unclear. CXCR3 is strongly implicated in coordinating the migration of SLO-originating ASCs into inflamed sites (52–56, 83), and CXCR3 is reported to promote differentiation of GCB cells into ASCs (40). Further, CXCR3 may direct the interstitial positioning of ASCs in the lung to microenvironments that promote ASC survival following IAV infection, potentially through facilitating access to niches rich in ASC trophic factors such as BlyS, APRIL and IL-6 (84). Notably, CXCR3 expression is higher on class-switched IgG^+^ ASCs compared to IgM^+^ ASCs, and this may result in differential homing and/or localization potential of these Ig-producing ASC populations in the lung. Interestingly, single-cell BCR sequencing analyses have revealed clonal overlap between ASCs and GCB cells in the lung (47), indicating a proportion of lung ASCs are derived from pulmonary GCB cells. In line with this phenomenon, the reduction in frequency of class-switching in CXCR3-deficient NP-specific GCB cells in pulmonary GCs observed in the present study may contribute to the impaired generation of IgG^+^ ASCs in the lung at d14-15 postinfection with IAV. Further analyses are required to evaluate the role of CXCR3 in differentiation of ASCs from pulmonary GCB cells and to specifically investigate whether CXCR3 regulates SLO-generated ASC recruitment from circulation into the inflamed lung. Crossing *Cd23^Cre/+^* and *Cd23^Cre/+^hCXCR3^fl/fl^* mice to Blimp1-reporter mice could assist these investigations (85), enabling flow cytometric analysis of ASC differentiation among CXCR3-sufficient and -deficient lung GCB cells and sort-transfer studies of ASCs in IAV infected hosts. Overlaying these analyses with assessment of different Ig isotypes in responding B cell populations, including GCB cells, MBCs, and ASCs, would also be valuable for developing understanding of CXCR3-mediated regulation of specific Ig-producing subsets, as different Ig isotypes can provide important contributions to protective immune mechanisms against IAV (33).

Overall, we demonstrate CXCR3 is prominently expressed by multiple responding B cell populations across several anatomical compartments following IAV infection, and it is significantly enriched on GCB cells and swB_mem_ cells at the site of infection in the lung relative to their counterparts in SLOs. We show that B cell–expressed CXCR3 drives efficient generation of GCB cells in iBALT following IAV infection in both isolated and competitive settings. Therefore, the findings position CXCR3 as a key molecular requirement for generation of pulmonary GCB cell responses in iBALT following respiratory viral infection with IAV.

## Methods

### Mice.

B6 (C57Bl/6 J) and Ly5.1 (C57Bl/6 J.SJL-Ptprca) mice were purchased from Ozgene, formerly the Animal Resource Centre, (WA, Australia). CXCR3 internal ribosome entry site bicistronic enhanced GFP reporter mice [*CIBER,* B6.129S4-*Cxcr3^tm1Arsa^*/SoghJ (49)] were obtained from Andrew D. Luster (Massachusetts General Hospital, Harvard University, USA) and maintained in-house by crossing hemizygous males with homozygous females. *Cd23^Cre/+^* mice [B6.Cg-Tg(*Fcer2a*-cre)5Mbu/J (86)] and *Cd23^Cre/+^hCXCR3^fl/fl^* mice (40) were obtained from Kim L. Good-Jacobson (Monash University, Vic, Australia). *Cd23^Cre/+^* mice were maintained in-house by crossing *Cd23^Cre/+^* mice with *Cd23^Cre^*-transgene negative mice. *Cd23^Cre/+^hCXCR3^fl/fl^* mice were maintained in-house by crossing mice homozygous or hemizygous for the floxed *hCXCR3* allele (*hCXCR3^fl/fl^* or *hCXCR3^fl/y^*) and heterozygous for the *Cd23^Cre^* transgene with *Cd23^Cre^* transgene-negative *hCXCR3^fl/fl^* or *hCXCR3^fl/y^* mice. *Cd23^Cre/+^* heterozygous mice (CD45.2) were crossed in-house with Ly5.1 (CD45.1) mice to generate CD45.1/CD45.2^+^ *Cd23^Cre/+^* mice. MD4 mice [C57BL/6-Tg(IghelMD4)4Ccg/J (65)] on a Ly5.1 background were obtained from Robert Brink (Garvan Institute of Medical Research, NSW, Australia) and maintained in-house by crossing heterozygous males with Ly5.1 females. Aged-matched female mice at least 6 wk of age were used in experiments. All mice were housed in specific-pathogen free, temperature-controlled conditions on a 12 h:12 h light:dark cycle in the Helen Mayo Animal Facility or the Commonwealth Scientific and Industrial Research Organisation Facility at Adelaide University. All mice were provided chow and water ad libitum. Mice were humanely killed by CO_2_ asphyxiation. All murine experimental procedures and breeding protocols were approved by the Animal Ethics Committee at Adelaide University.

### B Cell Isolation and Adoptive Cell Transfer.

Naïve B cells for adoptive transfer were isolated from single-cell splenocyte suspensions using EasySep Mouse B Cell Isolation Kit (STEMCELL Technologies) according to manufacturers’ instructions. Purity of isolated B cells was routinely >90%. Flow cytometry was used to confirm congenically distinct B cells isolated from *Cd23^Cre/+^*(CD45.1/CD45.2) and *Cd23^Cre/+^hCXCR3^fl/fl^* (CD45.2) mice were mixed in equal parts prior to intravenous adoptive cell transfer into MD4 recipient mice. A total of 15 to 30 × 10^6^ naïve B cells were injected into MD4 recipient mice via the lateral tail vein in a volume of 200 to 250 µL of endotoxin-free PBS using a 29-gauge insulin syringe (BD Biosciences). MD4 recipient mice were then rested overnight and infected the following day with IAV.

### Production of IAV and Intranasal Infection.

A/Hong Kong/1/1968 (H3N2) IAV (x31) stocks were prepared by growing IAV in the allantoic cavity of 10-day-old embryonated chicken eggs for 48 h at 37 °C. Eggs were then chilled at 4 °C overnight and the allantoic fluid was harvested, pooled, clarified, aliquoted, and stored at −80 °C. Concentration of x31 was determined by 50% tissue culture infectious dose (TCID_50_) assays using Madin-Darby Canine Kidney cells and chicken red blood cells. TCID_50_ values for IAV stocks were calculated using the Reed and Muench technique (87).

For IAV infection, mice were anesthetized with 60 µg/g of pentobarbitone injected intraperitoneally. Anesthetized mice were held in an upright position and a dose of 10 TCID_50_ x31 in 32 µL endotoxin-free PBS administered intranasally via dropwise pipetting. IAV-infected mice were monitored and weighed daily in accordance with ethical obligations approved by the Animal Ethics Committee at Adelaide University.

### Intravascular Labeling.

To enable ex vivo flow cytometric discrimination between cells in circulation and cells within the parenchyma of tissues, intravascular lymphocytes were labeled by intravenous injection with 3 µg of fluorophore-conjugated antibody (αCD45: clone 30-F11, αCD45.2: clone A20) in 200 µL of endotoxin-free PBS 3 min prior to humane killing and downstream analysis.

### Murine Tissue Processing and Sample Collection.

#### Peripheral blood (PB).

PB was collected by cardiac puncture and immediately dispensed into heparinized Vacutainer blood collection tubes (BD Biosciences). Up to 400 μL of PB was then mixed with 10 mL prewarmed Mouse Red Cell Lysis Buffer (MRCLB, 139.5 mM NH_4_Cl, 17 mM Tris-HCl, pH 7.2) and incubated at 37 °C for 20 to 25 min with occasional mixing by inversion. Cell suspensions were then washed extensively with ice-cold PBS.

#### Bronchoalveolar lavage fluid (BALF).

To harvest BALF, the tracheas of humanely killed mice were surgically exposed, and a small superficial incision was made approximately halfway along the length of the trachea, avoiding completely severing the trachea. The end of an Insyte Autoguard Catheter (BD Biosciences), attached to a 1 mL Tuberculin Syringe (BD Biosciences) loaded with 1 mL of PBS supplemented with 5 mM EDTA, was inserted into the lumen of the trachea pointing toward the lungs. The airways were then gently washed 2 to 3 times with the same 1 mL of PBS/EDTA by repeatedly plunging and redrawing the syringe. When required, supernatants from BALF samples were collected into tubes, supplemented with a 1X Protease Inhibitor Cocktail (Sigma-Aldrich) and stored at −80 °C until downstream analysis.

#### Mediastinal LNs (mLNs), spleens, lungs, mesenteric LNs (mesLN), and Peyer’s patches (PP).

To prepare single-cell suspensions, mLNs, spleens, lungs, mesLNs, and PP were submerged in digest media (DMEM with 5 to 10% FCS (Sigma-Aldrich), 10 mM HEPES (Gibco), 100 U/mL Penicillin (Life Technologies), 100 μg/mL Streptomycin (Life Technologies), 2.5 mM calcium chloride, 2.5 mM magnesium chloride, 30 U/mL DNase I [Sigma-Aldrich) and 1 mg/mL Collagenase IA (Sigma-Aldrich)] in 5 mL yellow cap tubes (Techno Plas), mechanically dissociated by mincing with surgical scissors, and digested at 37 °C with shaking for 20 to 30 min. Lung samples were digested for 45 to 60 min with trituration using a 1 mL pipette every 15 to 20 min. Where ex vivo flow cytometric analysis of CD138 was to be performed, the mLNs and spleens were not digested and instead mechanically dissociated in PBS by pressing the organs through 70 µm nylon filters (Corning) with the flat end of a plunger from a 3 mL syringe (BD Biosciences). Digested lung tissue homogenates were filtered through 70 µm nylon filters (Corning). Cell pellets for spleen and lung samples were resuspended in 5 mL prewarmed MRCLB, incubated at 37 °C for 5 min and washed in PBS. When required, tissue supernatants were collected into fresh tubes and supplemented with Protease Inhibitor Cocktail (Sigma-Aldrich). Tissue supernatants were stored at −80 °C until downstream analysis.

### Flow cytometry.

For flow cytometric analyses, up to 2 × 10^6^ live cells were plated into 96-well U-bottom (round-bottom) or V-bottom trays (Corning or Thermo Fisher). For flow cytometric analyses, antibodies and reagents used for staining cells are listed in *SI Appendix*, Table S1. Incubations were performed in the dark at 4 °C unless stated otherwise. Cells were first stained with Fixable Viability Stain 780 (BD Biosciences) diluted 1/1,000 in PBS for 15 min at room temperature in the dark. Cells were washed with stain buffer [PBS supplemented with 1% (w/v) bovine serum albumin (BSA, Bovogen Biologicals) and 0.04% (w/v) sodium azide (Australian Chemical Reagents)]. Fc Receptors were blocked by resuspending in stain buffer containing 200 µg/mL rat γ-globulin (rγg, Rockland) and incubating at room temperature for 10 to 15 min. For IAV NP-specific B cell analysis, cells were resuspended in stain buffer containing 0.625 µg/mL IAV x31 recombinant his-tagged NP (rNP, SinoBiological) and 200 µg/mL rγg, incubated for 25 to 30 min, washed with stain buffer and resuspended in stain buffer containing 200 µg/mL rγg. Cells were then stained with fluorophore-labeled and biotinylated antibodies against cell surface antigens by adding antibody cocktails prepared in Brilliant Stain Buffer Plus (BD Biosciences) directly to the existing stain buffer containing 200 µg/mL rγg and incubating for 30 to 60 min. To detect biotinylated antibodies, cells were incubated with fluorophore-conjugated streptavidin diluted in Brilliant Stain Buffer (BD Biosciences) for 15 to 20 min. For staining intracellular immunoglobulin isotypes, the BD Cytofix/Cytoperm kit (BD Biosciences) was used as per manufacturers’ instructions. Cells were then washed and fixed in PBS containing 1% (w/v) paraformaldehyde and stored at 4 °C before acquisition on a BD LSR Fortessa flow cytometer. Data were analyzed with FlowJo Software Version 10 (BD Biosciences).

### Ex vivo chemotaxis assays.

Bulk single-cell suspensions from whole mLNs or spleens were prepared by mechanical dissociation as described above and rested in warm complete RPMI for 45 min at 37 °C, 5% CO_2_. Rested cells were then harvested and washed extensively with prewarmed chemotaxis buffer [RPMI 1640 (Gibco) supplemented with 25 mM HEPES (Gibco) and 0.5% (w/v) BSA] and resuspended at 2 × 10^7^ cells/mL. The bottom chamber of 24-well transwell plates (Corning) was loaded with 600 µL/well of prewarmed chemotaxis buffer containing 500 ng/mL synthetically produced murine CXCL11 (the late Ian Clark-Lewis, University of British Columbia, Canada). 100 µL of cell suspension (2 × 10^6^ cells) were then loaded into the upper chamber permeable supports (5 µm polycarbonate membrane). No chemokine control wells were loaded with 600 µL/well of prewarmed chemotaxis buffer. Plates were then incubated for 2 h at 37 °C, 5% CO_2_. Subsequently, the permeable supports were removed from the 24-well transwell plates and the underside of the polycarbonate membrane gently washed once with the liquid in the bottom of the wells. The pooled contents were then collected into individual wells of a 96-well V-bottom plate for subsequent flow cytometric analyses. Immediately prior to acquisition on the flow cytometer, 10 µL/well of CountBright Absolute Counting Beads (Invitrogen) were added to each sample. To assess chemotaxis, the migration index for cell populations of interest were calculated using the following formula:

$$\text{Migration}\;\text{Index}=\frac{\text{Number}\;\text{of}\;\text{migrated}\;\text{cells}\;\text{in}\;\text{sample}÷\text{Number}\;\text{of}\;\text{beads}\;\text{in}\;\text{sample}}{\text{Number}\;\text{of}\;\text{migrated}\;\text{cells}\;\text{in}\;\text{no}\;\text{chemokine}\;\text{control}\;\left(\text{avg}\right)÷\text{Number}\;\text{of}\;\text{beads}\;\text{in}\;\text{no}\;\text{chemokine}\;\text{control}\;(\text{avg})}.$$

### Multianalyte chemokine analysis.

To simultaneously assess the abundance of multiple cytokines and chemokines in samples from IAV-infected mice, a LEGENDplex bead-based immunoassay (BioLegend) was employed. A LEGENDplex Custom Mouse 12-plex Panel (BioLegend) was designed to analyze the concentration of IFNα, IFNβ, IFNγ, TNFα, IL-6, IL-10, CCL2, CCL20, CXCL9, CXCL10, CXCL12, and CXCL13 in supernatants isolated from BALF, mLNs, spleens, and lungs. The assay was performed according to the manufacturers’ guidelines. Data were analyzed using LEGENDplex data analysis software (BioLegend).

### Data analysis and statistics.

Statistical analyses and data processing was performed using GraphPad Prism version 10 software (Dotmatics). Statistical tests were two-sided and applied as detailed in the figure legends. Statistical significance was denoted as follows: *P < 0.05, **P < 0.01, ***P < 0.001, ****P < 0.0001.

## Supplementary Material

Appendix 01 (PDF)

## Data Availability

Study data are included in the article and/or *SI Appendix*.
