# Supplementary material for: B cell–intrinsic CXCR3 drives efficient generation of ectopic pulmonary germinal center responses to influenza A virus infection
Source: Proc Natl Acad Sci U S A. 2026 Jun 30;123(27):e2535787123. doi: 10.1073/pnas.2535787123 (PMC13342993; doi:10.1073/pnas.2535787123)
Supplement: Supplementary file 1 — Appendix 01 (PDF) [file pnas.2535787123.sapp.pdf]

Supplementary Figures, Figure Legends and Tables

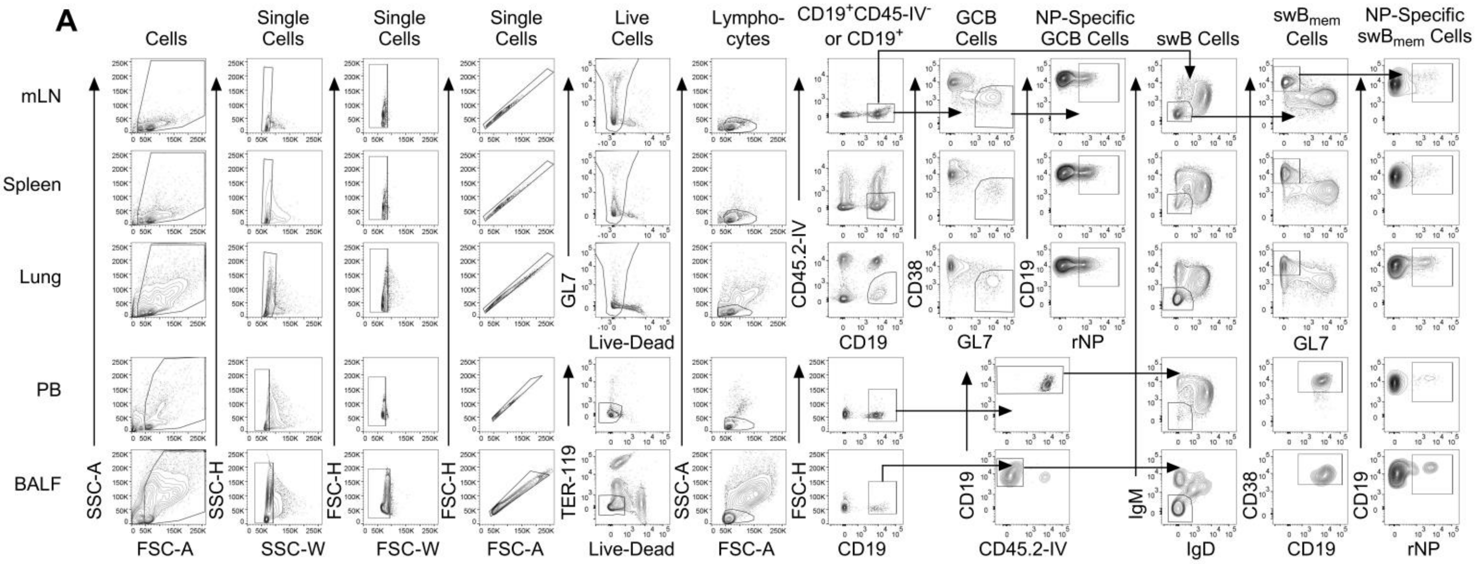

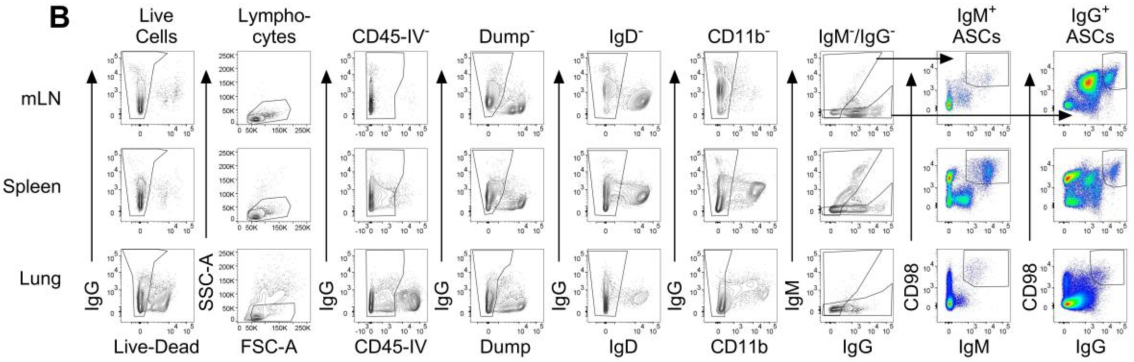

Supplementary Figure 1: Gating strategies for identification of responding B cell populations in CIBER mice. CIBER mice were infected with 10 $TCID_{50}$ x31 IAV and the mLN, spleen, lung, PB and BALF were harvested, after i.v-labelling, at multiple timepoints post-infection for flow cytometric analysis. (**A**) Representative gating strategies for identification of the indicated cell populations in each anatomical location at d14 post-infection. (**B**) Representative gating strategies for identification of IgM^+ and IgG^+ ASCs in the mLN, spleen and lung on d14 post-infection. Live cells are pre-gated on single cells using the gating strategy represented in Supplementary Figure 1 A.

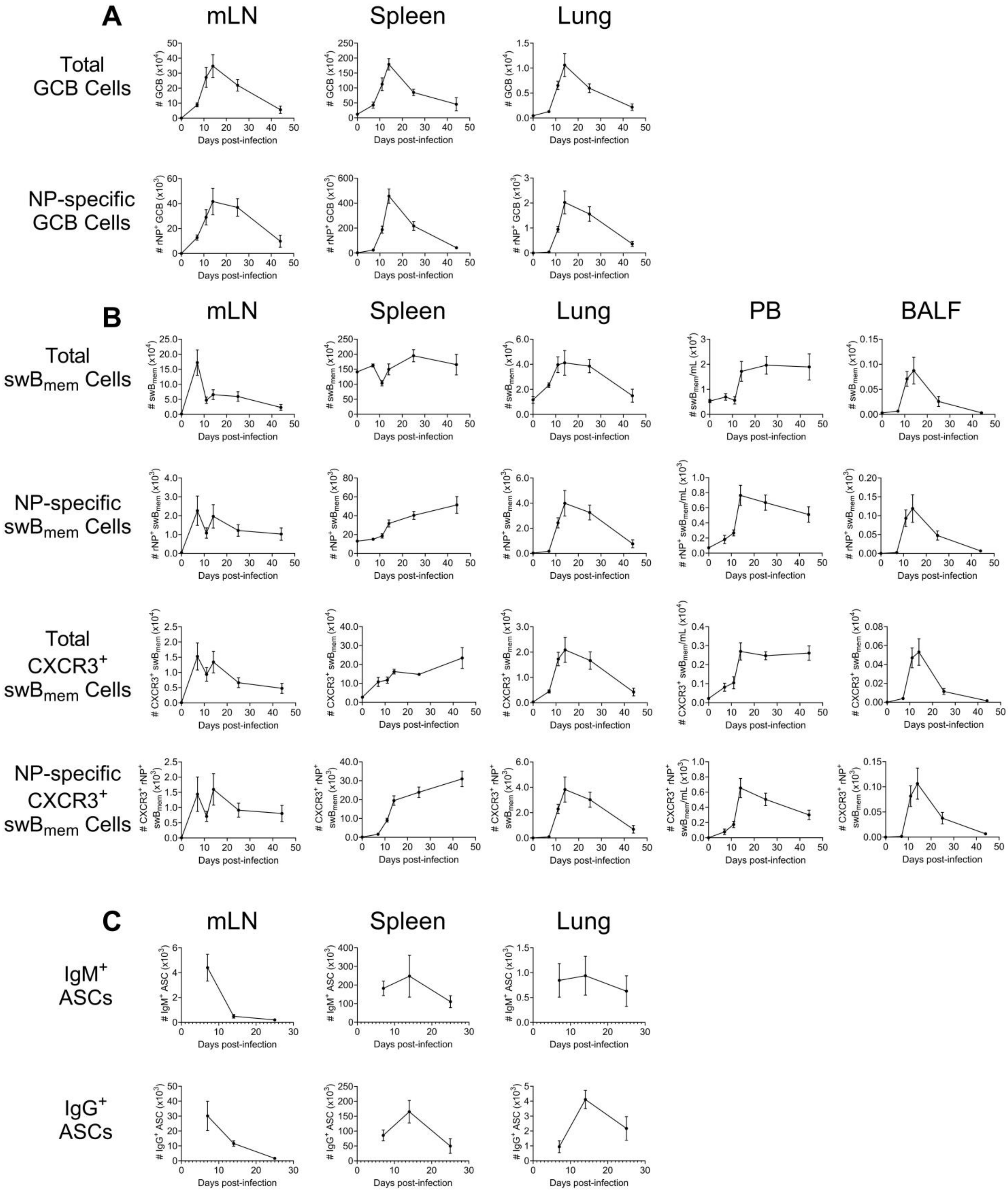

A
mLN
Spleen
Lung
Total GCB Cells
GCB (x10^4)
NP-specific GCB Cells
rNP$^+$ GCB (x10^3)
Days post-infection
B
mLN
Spleen
Lung
PB
BALF
Total swB$_{mem}$ Cells
swB$_{mem}$ (x10^4)
swB$_{mem}$/mL (x10^4)
NP-specific swB$_{mem}$ Cells
rNP$^+$ swB$_{mem}$ (x10^3)
rNP$^+$ swB$_{mem}$/mL (x10^3)
Total CXCR3$^+$ swB$_{mem}$ Cells
CXCR3$^+$ swB$_{mem}$ (x10^4)
CXCR3$^+$ swB$_{mem}$/mL (x10^4)
NP-specific CXCR3$^+$ swB$_{mem}$ Cells
CXCR3$^+$ rNP$^+$ swB$_{mem}$ (x10^3)
CXCR3$^+$ rNP$^+$ swB$_{mem}$/mL (x10^3)
Days post-infection
C
mLN
Spleen
Lung
IgM$^+$ ASCs
IgM$^+$ ASC (x10^3)
IgG$^+$ ASCs
IgG$^+$ ASC (x10^3)
Days post-infection

Supplementary Figure 2: Kinetics and magnitude of GCB cell, swB$_{mem}$ cell and ASC responses following IAV infection. CIBER mice were infected with 10 TCID$_{50}$ x31 IAV and the mLN, spleen, lung, PB and BALF were harvested on day 7, 11, 14, 25 and 44 post-infection, after i.v-labelling, for flow cytometric analysis. (**A**) Kinetics of total GCB cells and NP-specific GCB cells. (**B**) Kinetics of total swB$_{mem}$ cells, NP-specific swB$_{mem}$ cells, total CXCR3^{+} swB$_{mem}$ cells and NP-specific CXCR3^{+} swB$_{mem}$ cells. (**C**) Kinetics of IgM^{+} and IgG^{+} ASC responses. Data presented as mean ± SEM. n = 4-9 mice/time point, pooled from 2-3 independent experiments.

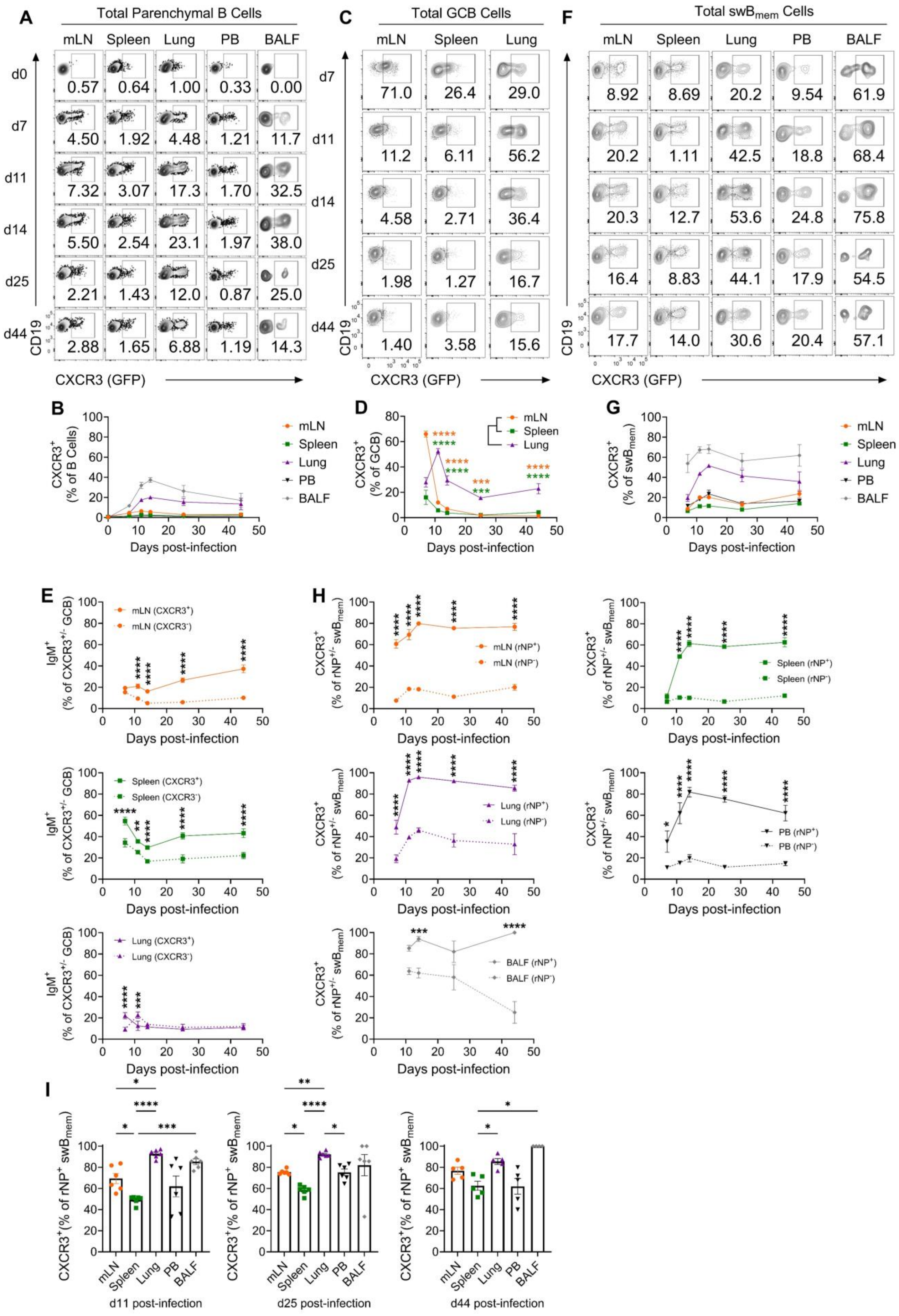

A
Total Parenchymal B Cells
mLN Spleen Lung PB BALF
d0 0.57 0.64 1.00 0.33 0.00
d7 4.50 1.92 4.48 1.21 11.7
d11 7.32 3.07 17.3 1.70 32.5
d14 5.50 2.54 23.1 1.97 38.0
d25 2.21 1.43 12.0 0.87 25.0
d44 2.88 1.65 6.88 1.19 14.3
CD19
CXCR3 (GFP)
C
Total GCB Cells
mLN Spleen Lung
d7 71.0 26.4 29.0
d11 11.2 6.11 56.2
d14 4.58 2.71 36.4
d25 1.98 1.27 16.7
d44 1.40 3.58 15.6
CD19
CXCR3 (GFP)
F
Total swB$_{mem}$ Cells
mLN Spleen Lung PB BALF
8.92 8.69 20.2 9.54 61.9
20.2 1.11 42.5 18.8 68.4
20.3 12.7 53.6 24.8 75.8
16.4 8.83 44.1 17.9 54.5
17.7 14.0 30.6 20.4 57.1
CD19
CXCR3 (GFP)
B
CXCR3$^+$ (% of B Cells)
Days post-infection
mLN Spleen Lung PB BALF
D
CXCR3$^+$ (% of GCB)
Days post-infection
mLN Spleen Lung
G
CXCR3$^+$ (% of swB$_{mem}$)
Days post-infection
mLN Spleen Lung PB BALF
E
IgM$^+$ (% of CXCR3$^{+/-}$ GCB)
mLN (CXCR3$^+$)
mLN (CXCR3$^-$)
Spleen (CXCR3$^+$)
Spleen (CXCR3$^-$)
Lung (CXCR3$^+$)
Lung (CXCR3$^-$)
Days post-infection
H
CXCR3$^+$ (% of rNP$^{+/-}$ swB$_{mem}$)
mLN (rNP$^+$)
mLN (rNP$^-$)
Spleen (rNP$^+$)
Spleen (rNP$^-$)
Lung (rNP$^+$)
Lung (rNP$^-$)
PB (rNP$^+$)
PB (rNP$^-$)
BALF (rNP$^+$)
BALF (rNP$^-$)
Days post-infection
I
CXCR3$^+$(% of rNP$^+$ swB$_{mem}$)
mLN Spleen Lung PB BALF
d11 post-infection
d25 post-infection
d44 post-infection

Supplementary Figure 3: Kinetics of CXCR3 expression in B cells, GCB cells and swB_{mem} cells following IAV infection. CIBER mice were infected with 10 $TCID_{50}$ x31 IAV and the mLN, spleen, lung, PB and BALF were harvested on day 7, 11, 14, 25 and 44 post-infection, after i.v-labelling, for flow cytometric analysis. (**A**) Representative flow cytometry for identification of CXCR3-expressing B cells. Flow plots are pre-gated on live, $CD19^+$, parenchymal cells for the mLN, spleen, lung and BALF. In the PB, the flow plots were gated similarly, with the difference that cells were instead positive for the i.v-label. Representative gating strategies for identifying live, CD19, parenchymal or vascular B cells in each anatomical location are depicted in Supplementary Figure 1 A. (**B**) Quantification of (A). (**C**) Representative flow cytometry for longitudinal assessment of CXCR3 expression in total GCB cells. (**D**) Quantification of (C). (**E**) IgM positivity in $CXCR3^+$ or $CXCR3^-$ GCB cells. (**F**) Representative flow cytometry for longitudinal assessment of CXCR3 expression in total swB_{mem} cells. (**G**) Quantification of (F). (**H**) CXCR3 expression in NP-specific (rNP^+) or not NP-specific (rNP^-) swB_{mem} cells. (**I**) Quantification of Figure 1 E for statistical comparisons at d11, d25 and d44 post-infection. Data presented as mean ± SEM. n = 4-9 mice/time point, pooled from 2-3 independent experiments. Data in (D, E & H) were analysed by repeated measures two-way ANOVA (mLN, spleen, lung) or mixed-effects analysis (PB, BALF) with Bonferroni's multiple comparisons test. Data in (I) were analysed by repeated measures one-way ANOVA (or mixed-effects analysis for comparisons with BALF at d44 post-infection) with Bonferroni's multiple comparisons test. *$p < 0.05$, **$p < 0.01$, ***$p < 0.001$, ****$p < 0.0001$.

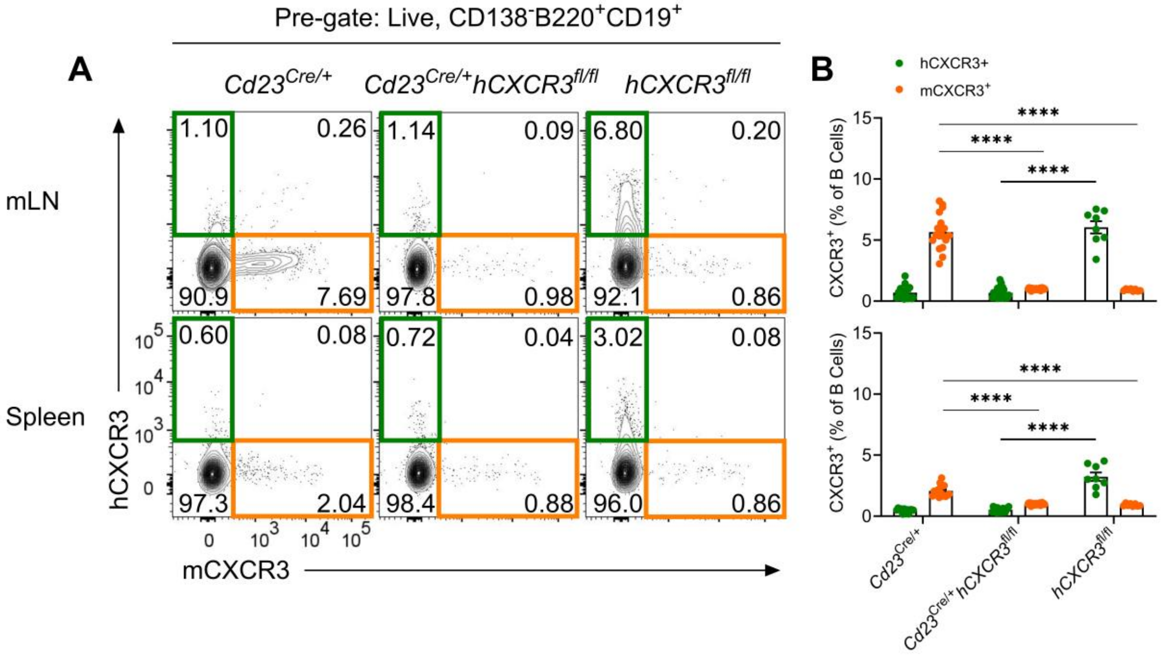

Pre-gate: Live, CD138-B220+CD19+
A
Cd23Cre/+
Cd23Cre/+hCXCR3fl/fl
hCXCR3fl/fl
mLN
Spleen
1.10
0.26
90.9
7.69
1.14
0.09
97.8
0.98
6.80
0.20
92.1
0.86
0.60
0.08
97.3
2.04
0.72
0.04
98.4
0.88
3.02
0.08
96.0
0.86
hCXCR3
mCXCR3
B
hCXCR3+
mCXCR3+
CXCR3+ (% of B Cells)
****

Supplementary Figure 4: *Cd23$^{Cre/+}$hCXCR3$^{fl/fl}$* mice provide a model to study CXCR3 deficiency in B cells. *Cd23$^{Cre/+}$*, *Cd23$^{Cre/+}$hCXCR3$^{fl/fl}$* and *Cd23$^{+/+}$hCXCR3$^{fl/fl}$* (*hCXCR3$^{fl/fl}$*) mice were infected with 10 $TCID_{50}$ x31 IAV and the mLN and spleen were harvested on d7 post-infection for flow cytometric analysis. (**A**) Representative flow cytometry for detection of human CXCR3 (hCXCR3) and murine CXCR3 (mCXCR3) expression on live, $CD138^{-}B220^{+}CD19^{+}$ cells from the mLN (top panel) and spleen (bottom panel). (**B**) Quantification of (A) in the mLN (top) and spleen (bottom). Data are presented as mean ± SEM. n = 8-22 mice/genotype, pooled from 2-4 independent experiments. Data in B were analysed by unpaired t-tests to compare hCXCR3 positivity on B cells between *Cd23$^{Cre/+}$hCXCR3$^{fl/fl}$* and *hCXCR3$^{fl/fl}$* mice and mCXCR3 positivity on B cells between *Cd23$^{Cre/+}$* and *Cd23$^{Cre/+}$hCXCR3$^{fl/fl}$* or *hCXCR3$^{fl/fl}$* mice. ****$p < 0.0001$.

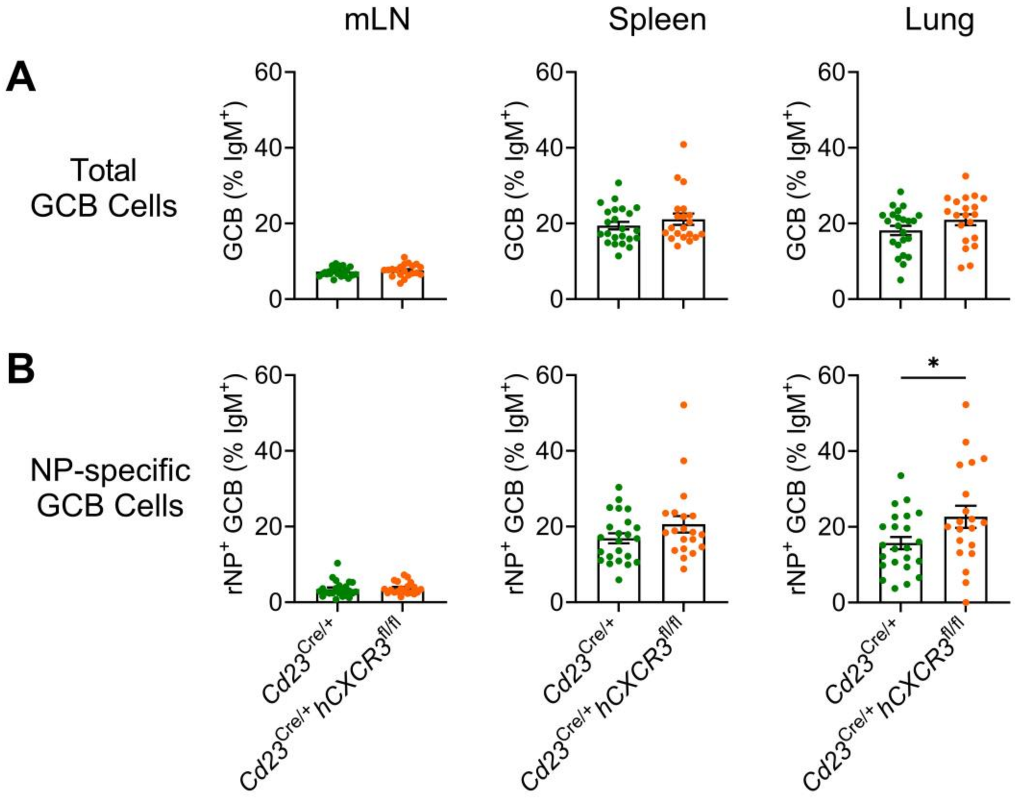
mLN
Spleen
Lung
A
Total
GCB Cells
GCB (% IgM+)
60
40
20
0
B
NP-specific
GCB Cells
rNP+ GCB (% IgM+)
*
Cd23Cre/+
Cd23Cre/+ hCXCR3fl/fl

Supplementary Figure 5: Lack of CXCR3 expression in B cells reduces frequency of class-switched GCB cells in ectopic GCs in the lung following IAV infection.

Cd23$^{Cre/+}$ and *Cd23$^{Cre/+}$hCXCR3$^{fl/fl}$* mice were infected with 10 $TCID_{50}$ x31 IAV and the mLN, spleen and lung were harvested on day 14-15 post-infection for flow cytometric analysis. (**A-B**) Quantification of IgM positivity in (**A**) total GCB cells and (**B**) NP-specific GCB B cells in the mLN, spleen and lung. Data are presented as mean ± SEM. (A-B) n = 20-23 mice/group, pooled from 8 independent experiments. Data in (A-B) were analysed by unpaired t-test. *$p < 0.05$.

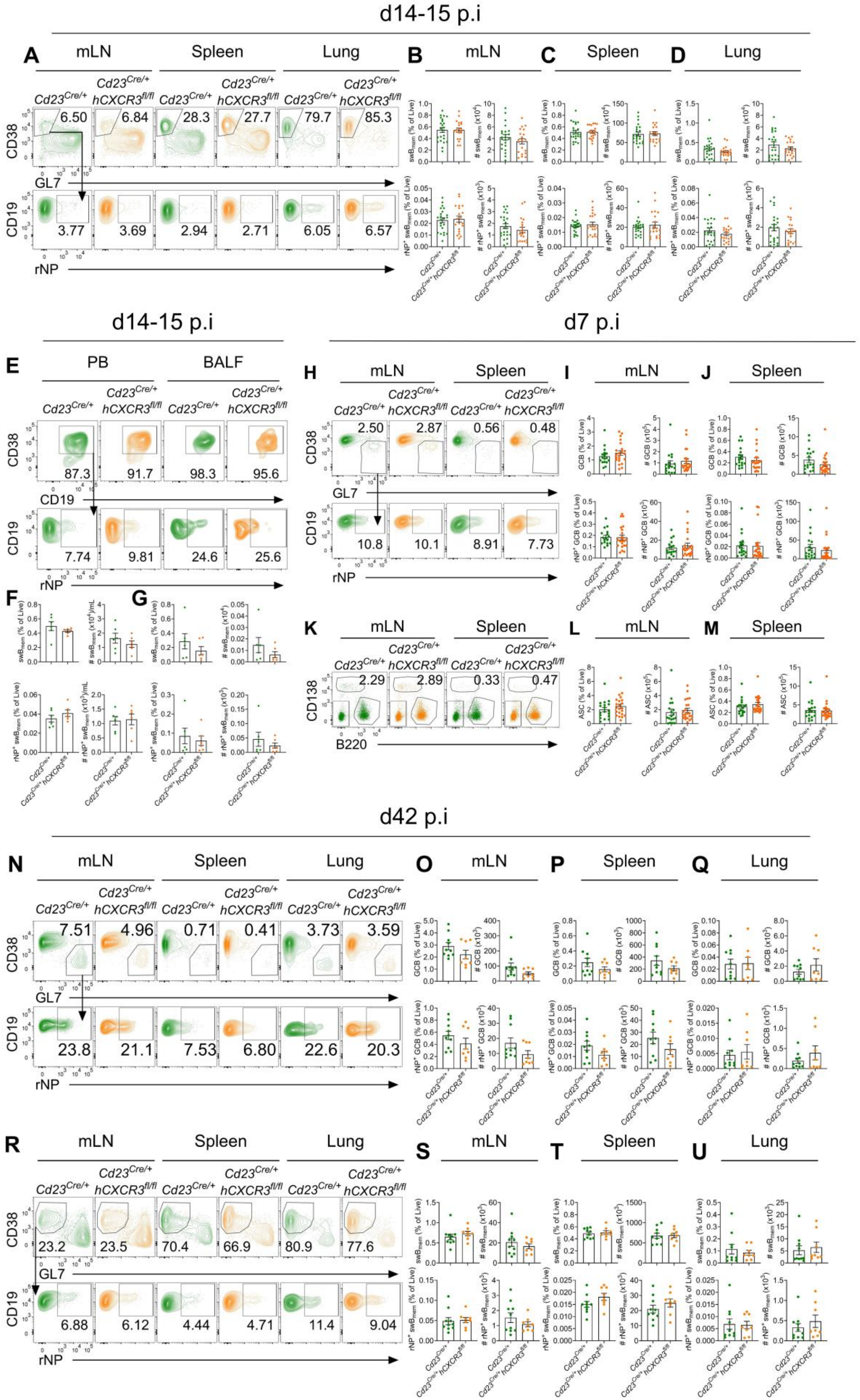

d14-15 p.i
A
mLN
Spleen
Lung
B
mLN
C
Spleen
D
Lung
Cd23Cre/+
Cd23Cre/+ hCXCR3fl/fl
CD38
GL7
CD19
rNP
6.50
6.84
28.3
27.7
79.7
85.3
3.77
3.69
2.94
2.71
6.05
6.57
d14-15 p.i
d7 p.i
E
PB
BALF
87.3
91.7
98.3
95.6
7.74
9.81
24.6
25.6
F
G
H
mLN
Spleen
2.50
2.87
0.56
0.48
10.8
10.1
8.91
7.73
I
mLN
J
Spleen
K
mLN
Spleen
CD138
B220
2.29
2.89
0.33
0.47
L
mLN
M
Spleen
d42 p.i
N
mLN
Spleen
Lung
7.51
4.96
0.71
0.41
3.73
3.59
23.8
21.1
7.53
6.80
22.6
20.3
O
mLN
P
Spleen
Q
Lung
R
mLN
Spleen
Lung
23.2
23.5
70.4
66.9
80.9
77.6
6.88
6.12
4.44
4.71
11.4
9.04
S
mLN
T
Spleen
U
Lung

Supplementary Figure 6: Lack of CXCR3 expression in B cells does not impact generation of swB$_{mem}$ cells at d14-15 post-infection, early GCB cells or ASCs at d7 post-infection, or late-phase GCB cells or swB$_{mem}$ cells at d42 post-infection, following primary IAV infection. (**A-R**) *Cd23$^{Cre/+}$* and *Cd23$^{Cre/+}$hCXCR3$^{fl/fl}$* mice were infected with 10 TCID$_{50}$ x31 IAV and the mLN, spleen (A-D, H-U), lung (A-D, N-U), PB and BALF (E-G) were harvested, with (A-G, N-U) or without (H-M) prior i.v-labelling, at d14-15 (E-G), d7 (H-M) or d42 (N-U) post-infection. (**A**) Representative flow cytometry for identification of total (top panel) and IAV NP-specific (bottom panel) swB$_{mem}$ cells in the mLN, spleen and lung at d14-15 post-infection. Flow plots for total swB$_{mem}$ cells are pre-gated on live, B220$^+$CD19$^+$CD45-IV$^-$IgD$^-$IgM$^-$ cells. (**B-D**) Quantification of (A) by percentage of total live cells (left) and number (right) for total (top panel) and IAV NP-specific (bottom panel) swB$_{mem}$ cells in the (**B**) mLN, (**C**) spleen and (**D**) lung. (**E**) Representative flow cytometry for identification of total (top panel) and IAV NP-specific (bottom panel) swB$_{mem}$ cells in the PB and BALF at d14-15 post-infection. Flow plots for total swB$_{mem}$ cells are pre-gated on live, TER119$^-$CD19$^+$, CD45-IV$^+$ (PB) or CD45-IV$^-$ (BALF), IgD$^-$IgM$^-$ cells. (**F-G**) Quantification of (E) by percentage of total live cells (left) and number (right) for total (top panel) and IAV NP-specific (bottom panel) swB$_{mem}$ cells in the (**F**) PB and (**G**) BALF. (**H**) Representative flow cytometry for identification of total (top) and IAV NP-specific (bottom) GCB cells at d7 post-infection. Flow plots in the top panel are pre-gated on live, CD138$^-$ B220$^+$CD19$^+$ cells. (**I-J**) Quantification of (H) by percentage of total live cells (left) and number (right) for total (top panel) and IAV NP-specific (bottom panel) GCB cells in the (**I**) mLN and (**J**) spleen. (**K**) Representative flow cytometry for identification of ASCs at d7 post-infection. Flow plots are pre-gated on live cells. (**L-M**) Quantification of (K) by percentage of total live cells (left) and number (right) for ASCs in the (**L**) mLN and (**M**) spleen. (**N**) Representative flow cytometry for identification of total (top panel) and IAV NP-specific (bottom panel) GCB cells in the mLN, spleen and lung at d42 post-infection. Flow plots for total GCB cells are pre-gated on live, B220$^+$CD19$^+$CD45-IV$^-$ cells. (**O-Q**) Quantification of (N) by percentage of total live cells (left) and number (right) for total (top) and IAV NP-specific (bottom) GCB cells in the (**O**) mLN, (**P**) spleen and (**Q**) lung. (**R**) Representative flow cytometry for identification of total (top panel) and IAV NP-

specific (bottom panel) swB_{mem} cells in the mLN, spleen and lung at d42 post-infection. Flow plots for total swB_{mem} cells are pre-gated on live, $B220^{+}CD19^{+}CD45\text{-}IV^{-}IgD^{-}IgM^{-}$ cells. (**S-U**) Quantification of (R) by percentage of total live cells (left) and number (right) for total (top) and IAV NP-specific (bottom) swB_{mem} cells in the (**S**) mLN, (**T**) spleen and (**U**) lung. Data are presented as mean ± SEM. (A-D) n = 20-23 mice/group, pooled from 8 independent experiments. (E-G) n = 6 mice/group, pooled from 2 independent experiments. (H-M) n = 17-22 mice/group, pooled from 4 independent experiments. (N-U) n = 8-10 mice/group, pooled from 2 independent experiments.

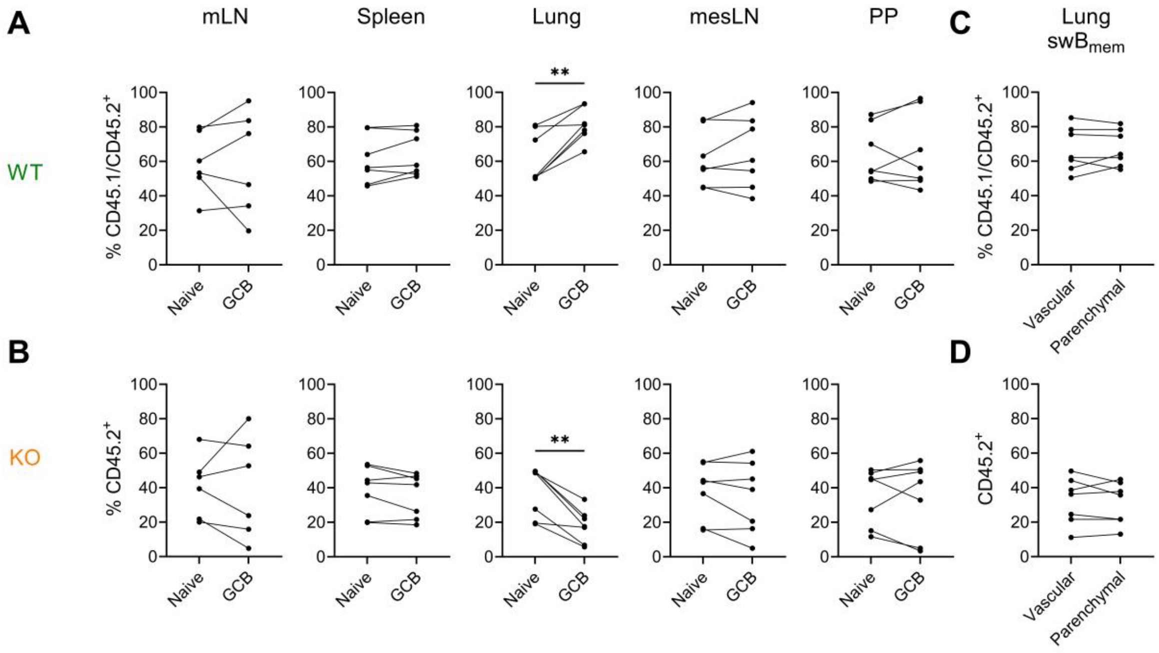

A
B
C
D
mLN
Spleen
Lung
mesLN
PP
Lung swB$_{mem}$
WT
KO
% CD45.1/CD45.2^{+}
% CD45.2^{+}
CD45.2^{+}
**
Naive
GCB
Vascular
Parenchymal

Supplementary Figure 7: Lack of CXCR3 expression in B cells restricts their competitive fitness in ectopic pulmonary GC responses following IAV infection but does not impact lung infiltration of swB$_{mem}$ cells. B cells were isolated from the spleens of naïve *Cd23$^{Cre/+}$* (CD45.1/CD45.2) and *Cd23$^{Cre/+}$hCXCR3$^{fl/fl}$* (CD45.2) mice, mixed, and transferred intravenously to MD4 (CD45.1) hosts at doses of 15-30x10^6 total B cells. MD4 host mice were then intranasally infected with 10 TCID$_{50}$ x31 IAV the following day. On d14 post-infection, the mLN, spleen, lung, mesLN and PP were harvested from MD4 host mice for analysis. (**A-B**) The proportions of CD45.1/CD45.2$^+$ *Cd23$^{Cre/+}$* (WT) (**A**) or CD45.2$^+$ *Cd23$^{Cre/+}$hCXCR3$^{fl/fl}$* (KO) (**B**) cells among donor-derived naïve B cells and GCB cells in the mLN, spleen, lung, mesLN and PP of MD4 hosts. (**C-D**). The proportions of CD45.1/CD45.2$^+$ (WT, *Cd23$^{Cre/+}$*) (**C**) or CD45.2$^+$ (KO, *Cd23$^{Cre/+}$hCXCR3$^{fl/fl}$*) (**D**) cells among donor-derived vascular and parenchymal swB$_{mem}$ cells in the lung of MD4 hosts. The proportions for each cell population in each individual mouse are connected by lines. n = 6-7 mice/organ, pooled from 3 independent experiments. Data were analysed by paired t-tests. **$p < 0.01$.

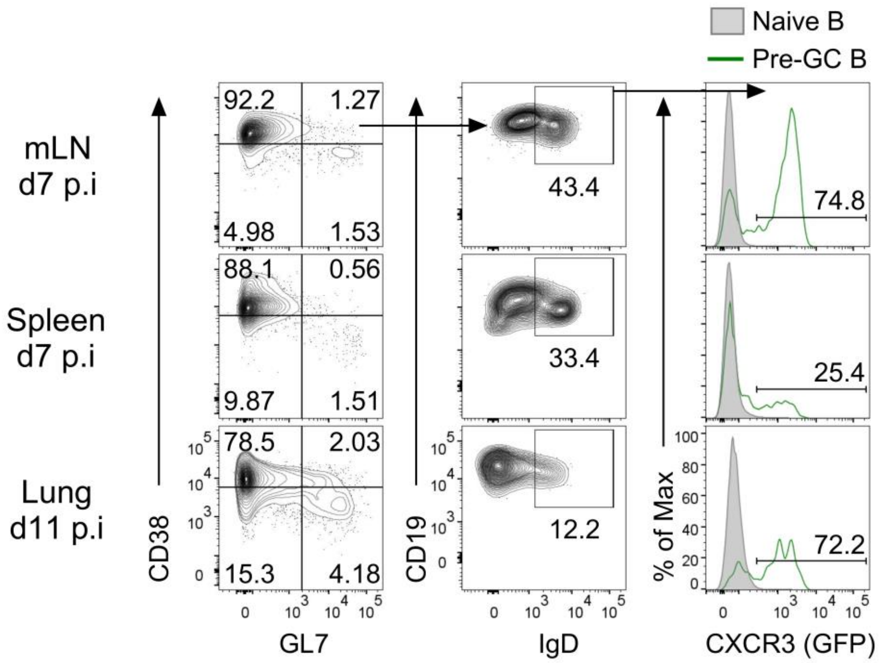

Naive B
Pre-GC B
mLN
d7 p.i
Spleen
d7 p.i
Lung
d11 p.i
92.2
1.27
4.98
1.53
88.1
0.56
9.87
1.51
78.5
2.03
15.3
4.18
43.4
33.4
12.2
74.8
25.4
72.2
CD38
GL7
CD19
IgD
% of Max
CXCR3 (GFP)

Supplementary Figure 8: Pre-GC B cells express CXCR3. CIBER mice were infected with 10 $TCID_{50}$ x31 IAV and the mLN, spleen and lung were harvested on day 7 or 11 post-infection, after i.v-labelling, for flow cytometric analysis. (**A**) Representative flow cytometry for identification of Pre-GC B cells ($CD38^{+}GL7^{+}IgD^{+}$ B cells) and assessment of their CXCR3 expression. Flow plots on the left are pre-gated on live, $CD19^{+}CD45\text{-}IV^{-}$ cells. p.i = post-infection.

Supplementary Table 1: Antibodies and reagents for flow cytometry.

**Antibodies Against Cell Surface Molecules**					
**Specificity**	**Format**	**Isotype**	**Clone**	**Source**	**Working Concentration**
Mouse CD3ε	Fluorophore-conjugated	Armenian Hamster IgG1, κ	145-2C11	BD Biosciences	1.11-1.67 μg/mL
Mouse CD4	Fluorophore-conjugated	Rat IgG2a, κ	RM4-5	BD Biosciences	0.67 μg/mL
Mouse CD8α	Fluorophore-conjugated	Rat IgG2a, κ	53-6.7	BD Biosciences	0.67 μg/mL
Mouse CD11b	Fluorophore-conjugated	Rat IgG2b, κ	M1/70	BD Biosciences	0.67 μg/mL
Mouse CD19	Fluorophore-conjugated	Rat IgG2a, κ	1D3	BD Biosciences	0.67 μg/mL
Mouse CD38	Fluorophore-conjugated	Rat IgG2a, κ	90	BD Biosciences	0.67 μg/mL
Mouse CD45.1	Fluorophore-conjugated	Mouse IgG2a, κ	A20	BD Biosciences & BioLegend	0.67 μg/mL
Mouse CD45.2	Fluorophore-conjugated	Mouse IgG2a, κ	104	BD Biosciences & BioLegend	0.67 μg/mL
Mouse CD86	Fluorophore-conjugated	Rat IgG2a, κ	GL1	BD Biosciences & BioLegend	0.83 μg/mL
Mouse CD98	Fluorophore-conjugated	Rat IgG2a, κ	RL388	BD Biosciences & BioLegend	0.67 μg/mL
Mouse CD138	Fluorophore-conjugated	Rat IgG2a, κ	281-2	BD Biosciences	0.67 μg/mL
Mouse B220 (CD45R)	Fluorophore-conjugated	Rat IgG2a, κ	RA3-6B2	BD Biosciences & BioLegend	0.67 μg/mL

Antibodies Against Cell Surface Molecules (Supplementary Table 1, continued)					
**Specificity**	**Format**	**Isotype**	**Clone**	**Source**	**Working Concentration**
Erythrocytes	Fluorophore-conjugated	Rat IgG2b, κ	TER-119	BD Biosciences	0.67 µg/mL
Erythrocytes	Biotin-conjugated	Rat IgG2b, κ	TER-119	BioLegend	1.04 µg/mL
His Tag	Fluorophore-conjugated	Mouse IgG2a, κ	J095G46	BioLegend	0.03 µg/well
Mouse IgD	Fluorophore-conjugated	Rat IgG2a, κ	11-26c.2a	BD Biosciences	0.67 µg/mL
Mouse IgM	Fluorophore-conjugated	Rat IgG2a, κ	II/41	BD Biosciences	1.11 µg/mL
Mouse/Human T and B cell Activation Marker	Fluorophore-conjugated	Rat IgM, κ	GL7	BD Biosciences & BioLegend	2.22-2.78 µg/mL
Mouse CXCR3	Fluorophore-conjugated	Armenian Hamster IgG	CXCR3-173	BioLegend	1.11 µg/mL
Human CXCR3	Fluorophore-conjugated	Mouse IgG1, κ	1C6	BD Biosciences	5 µL/well
Mouse CXCR4	Fluorophore-conjugated	Rat IgG2b, κ	2B11	BD Biosciences	1.11 µg/mL
Mouse CXCR4	Biotin-conjugated	Rat IgG2b, κ	2B11	BD Biosciences	1.67 µg/mL

Antibodies Against Intracellular Immunoglobulins (Supplementary Table 1, continued)					
**Specificity**	**Format**	**Isotype**	**Clone**	**Source**	**Working Concentration**
Mouse IgM	Fluorophore-conjugated	Rat IgG2a, κ	eB121-15F9	Invitrogen/ eBioscience	1.11 µg/mL
Mouse IgG	Fluorophore-conjugated	Goat IgG	Polyclonal	SouthernBiotech	2.0 µg/mL
**Streptavidins (Supplementary Table 1, continued)**					
**Specificity**	**Format**	**Isotype**	**Clone**	**Source**	**Working Concentration**
Biotin	Fluorophore-conjugated Streptavidin	N/A	N/A	BD Biosciences & BioLegend	0.5-2.5 µg/mL
